# DualCMNet: a lightweight dual-branch network for maize variety identification based on multi-modal feature fusion

**DOI:** 10.3389/fpls.2025.1588901

**Published:** 2025-05-21

**Authors:** Xinhua Bi, Hao Xie, Ziyi Song, Jinge Li, Chang Liu, Xiaozhu Zhou, Helong Yu, Chunguang Bi, Ming Zhao

**Affiliations:** ^1^ College of Information Technology, Jilin Agricultural University, Changchun, China; ^2^ School of Electronic Information Engineering, Changchun University of Science and Technology, Changchun, China; ^3^ Jilin Zhongnong Sunshine Data Co., Ltd, Changchun, China

**Keywords:** maize variety classification, dual-branch network, lightweight network, hyperspectral and image data, multi-modal fusion

## Abstract

**Introduction:**

The accurate identification of maize varieties is of great significance to modern agricultural management and breeding programs. However, traditional maize seed classification methods mainly rely on single modal data, which limits the accuracy and robustness of classification. Additionally, existing multimodal methods face high computational complexity, making it difficult to balance accuracy and efficiency.

**Methods:**

Based on multi-modal data from 11 maize varieties, this paper presents DualCMNet, a novel dual-branch deep learning framework that utilizes a one-dimensional convolutional neural network (1D-CNN) for hyperspectral data processing and a MobileNetV3 network for spatial feature extraction from images. The framework introduces three key improvements: the HShuffleBlock feature transformation module for feature dimension alignment and information interaction; the Channel and Spatial Attention Mechanism (CBAM) to enhance the expression of key features; and a lightweight gated fusion module that dynamically adjusts feature weights through a single gate value. During training, pre-trained 1D-CNN and MobileNetV3 models were used for network initialization with a staged training strategy, first optimizing non-pre-trained layers, then unfreezing pre-trained layers with differentiated learning rates for fine-tuning.

**Results:**

Through 5-fold cross-validation evaluation, the method achieved a classification accuracy of 98.75% on the validation set, significantly outperforming single-modal methods. The total model parameters are only 2.53M, achieving low computational overhead while ensuring high accuracy.

**Discussion:**

This lightweight design enables the model to be deployed in edge computing devices, allowing for real-time identification in the field, thus meeting the practical application requirements in agricultural Internet of Things and smart agriculture scenarios. This study not only provides an accurate and efficient solution for maize seed variety identification but also establishes a universal framework that can be extended to variety classification tasks of other crops.

## 1 Introduction

Maize is one of the most significant food crops globally, and its variety identification plays a crucial role in agricultural production management, seed quality control, and breeding research (Tyczewska et al., 2018). Accurate variety identification not only helps protect intellectual property rights and regulate the seed market but also provides scientific evidence for breeding programs, thereby improving agricultural production efficiency and ensuring food security (Erenstein et al., 2022). However, due to the high morphological similarity among most maize varieties, while traditional techniques such as high-performance liquid chromatography, protein electrophoresis, and DNA molecular markers demonstrate high identification accuracy (Barrias et al., 2024; Li et al., 2024c; Moreno-Chamba et al., 2024), these methods generally have significant limitations including being destructive, time-consuming, operationally complex, and costly, making them challenging to implement on a large scale in practical production. Therefore, developing a rapid, accurate, and economical method for maize variety identification holds substantial theoretical value and practical significance.

In recent years, computer vision-based crop variety identification methods have garnered significant attention due to their advantages of being rapid, non-destructive, and cost-effective. Deep learning technology has achieved remarkable progress in image recognition. Feng et al. (2023) proposed a lightweight wheat seedling variety identification model, MssiapNet, which improved the MobileVit-XS network by incorporating scSE attention mechanism and IAP module, achieving 96.85% accuracy in identifying 29 varieties while maintaining model parameters at 29.70MB. Li et al. (2024a) developed an improved ResNet50-based maize seed identification model that introduced ResStage structure and efficient channel attention mechanism, achieving 91.23% accuracy in classifying six varieties while reducing model parameters by 40%. Yang et al. (2021) enhanced the VGG16 network structure by removing certain fully connected layers and adding depth cascade and batch normalization layers, achieving 96.7% accuracy in identifying 12 peanut varieties, with the method demonstrating good generalization performance in classifying seven maize varieties at 90.1% accuracy. Simultaneously, spectral technology has been widely applied in agricultural product quality evaluation (Zhang et al., 2021; Qin et al., 2024; Yang et al., 2024), composition content determination (Guo et al., 2023; Liang et al., 2024; Yang et al., 2024), and variety identification (Dong et al., 2024; Salehi et al., 2024; Bi et al., 2025). As a crucial identification tool, spectral analysis provides rich physicochemical component information of crop kernels, offering new technical pathways for variety identification. Zhang et al. (2024) proposed a spectral band selection method based on sparse band attention networks, achieving 95.20% classification accuracy using 50 selected bands on a hyperspectral dataset containing 20 maize varieties, showing a 1.35% improvement over full-band methods. Li et al. (2024b) developed a jujube variety traceability method based on near-infrared spectroscopy and one-dimensional CNN, achieving classification accuracies of 93.50%, 94.33%, and 94.25% using RBF, LSTM, and CNN respectively on a 4000-sample dataset, with CNN performing best under small-sample conditions, reaching 90.43% accuracy with 700 samples. Zhou et al. (2024) proposed an improved DenseNet-based maize seed identification method using near-infrared spectroscopy, enhancing DenseNet-121 through BCN, ACmix, and CBAM modules, achieving 99.33% accuracy in classifying five varieties while reducing model parameters to 0.97M. Yu et al. (2021) achieved 93.79% identification accuracy for 18 hybrid okra seed varieties using HSI (948.17-1649.20nm) combined with CNN technology. Wang and Song (2023) utilized hyperspectral imaging technology combined with deep learning methods to identify various sweet corn seeds, achieving classification accuracy exceeding 95% in both training and testing datasets. However, these unimodal methods still face significant challenges when dealing with visually similar varieties: image-based methods struggle to capture subtle differences between varieties, particularly in morphologically similar new breeding varieties, while spectral analysis, although providing rich physicochemical information, is susceptible to environmental factors and spectral overlap issues. The inherent limitations of single-modality methods have prompted researchers to explore more comprehensive solutions.

Based on the complementarity of image features and spectral information, multimodal data fusion methods show promising application prospects. Wang et al. (2016) combined hyperspectral data with image texture features and used least squares support vector machines to classify different maize seed varieties, achieving a classification accuracy of 88.89% based on two data fusion methods. Li et al. (2023) combined morphological and hyperspectral features using an improved one-dimensional CNN model to predict cotton seed vigor, obtaining a correlation coefficient of 0.9427 after fusing spectral and image features. Gong et al. (2024) proposed a Chinese medicine classification system based on spectral-image dual-modal fusion, combining one-dimensional laser-induced breakdown spectroscopy and two-dimensional image data, achieving 99.40% accuracy in classifying nine varieties of Lycium barbarum, while reducing model parameters to 2.95M. Tan et al. (2020) proposed a Dual-Attention Time-Aware Gated Recurrent Unit (DATA-GRU), which effectively addressed irregular sampling intervals and missing values in medical time series data through time-aware mechanisms and dual attention structures, achieving an AUC value of 91.9% in predicting in-hospital mortality on the MIMIC-III dataset. However, existing multimodal fusion methods still face the following challenges: 1) Information loss due to feature dimension mismatch, where existing methods often use simple feature concatenation or average pooling for fusion, failing to fully utilize complementary information from different modalities; 2) High computational complexity, as many fusion architectures employ complex attention mechanisms or multi-level feature fusion strategies, resulting in excessive model parameters and computational overhead, which hinders practical deployment; 3) Lack of optimization design for crop features, where generic feature fusion methods fail to fully consider the specificity of crop variety identification.

To address these challenges, this study proposes a novel dual-branch deep learning framework called DualCMNet. The framework incorporates three innovative designs: 1) A feature transformation module based on HShuffleBlock, which achieves feature dimension alignment and sufficient information interaction through grouped linear transformation and channel shuffling, effectively solving the dimension mismatch problem in multimodal feature fusion; 2) A lightweight gated fusion mechanism that dynamically adjusts feature weights using a single gate value, significantly reducing computational complexity while maintaining high performance; 3) An extensible spectral-spatial feature fusion framework that is not only applicable to maize variety identification but can also be extended to variety identification tasks for other crops such as rice and wheat. While improving crop variety identification accuracy, this study significantly reduces the model’s computational complexity, providing a feasible technical solution for practical application scenarios.

## 2 Material and methods

### 2.1 Experimental materials

#### 2.1.1 Sample preparation

**Figure 1:**
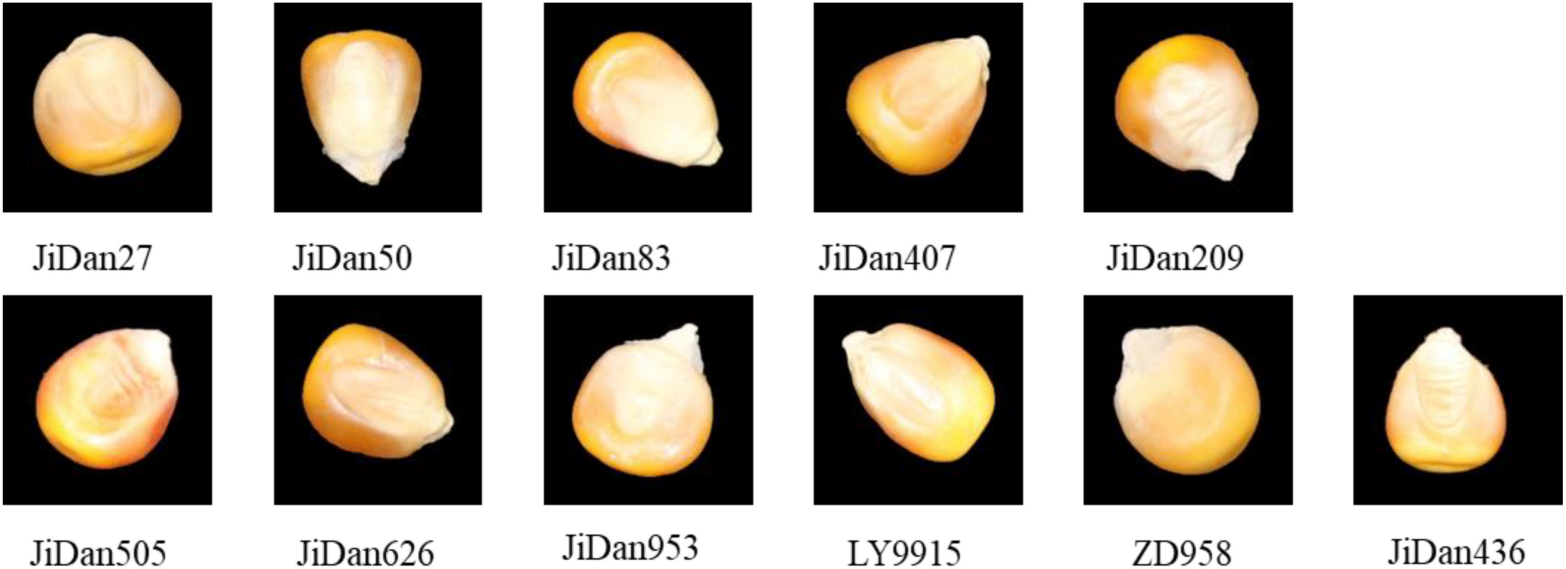
Maize kernel sample.

The maize kernels examined in this investigation were sourced from the Institute of Smart Agriculture, Jilin Agricultural University. The study incorporated 11 distinct cultivars: JiDan209, JiDan626, JiDan505, JiDan27, JiDan407, JiDan50, JiDan83, JiDan953, JiDan436, LY9915, and ZhengDan958 (**Figure 1**). To facilitate computational analysis, these varieties were sequentially coded numerically from 0 through 10 in the order listed. The selected cultivars constitute the primary maize varieties propagated throughout Jilin Province. Specifically, varieties in the JiDan series exhibit remarkable environmental adaptability and yield consistency, establishing them as predominant selections in Northeast China’s Spring Maize Region. ZhengDan958 demonstrates versatile adaptability across the Yellow-Huai-Hai Summer Maize Region, while LY9915 represents a significant cultivar extensively promoted throughout Northeastern regions. All kernel samples displayed yellow coloration, with several varieties exhibiting subtle reddish pigmentation on their surfaces. To maintain sample integrity and homogeneity, a meticulous manual selection process eliminated damaged, pest-affected, and foreign kernels, ensuring only fully developed and intact specimens were retained. Each varietal group comprised 1,000 individual kernels.

#### 2.1.2 Data acquisition and pre-processing

**Figure 2:**
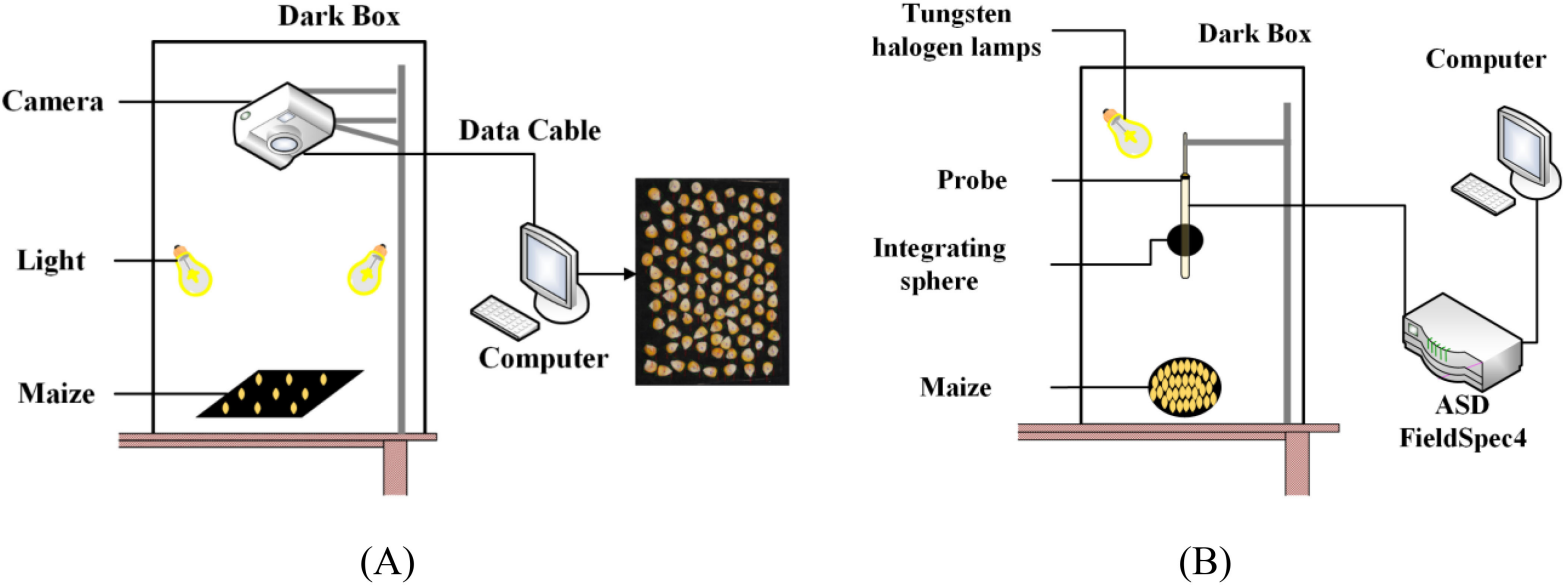
Real images and schematic diagram of maize kernel acquisition: **(A)** image data; **(B)** hyperspectral data.

This research employed a combination of RGB imaging and hyperspectral measurements to collect maize kernel data. RGB images were captured using a Canon EOS 1500D camera, with the acquisition system shown in **Figure 2A**. To ensure consistency in data collection conditions, a standardized acquisition platform was constructed in the laboratory, comprising a black background board, a vertically mounted camera, and two stable LED light sources. During the acquisition process, kernels of each variety were arranged in groups of 100 on the background board, with 10 groups of images captured per variety at a resolution of 6000×4000 pixels. The stable LED light sources and standardized acquisition environment effectively minimized the interference of external light sources on image quality.

Hyperspectral data were collected using a FieldSpec4 portable spectroradiometer (ASD Inc., USA), with the measurement system shown in **Figure 2B**. This device collected reflectance spectral data within the 350–2500 nm spectral range, featuring a wavelength accuracy of 0.5 nm and a repeatability precision of 0.1 nm. During data collection, the probe was maintained at a fixed distance of 10 cm from the sample surface, with a 20 W halogen lamp serving as the light source. To ensure data quality, the instrument was calibrated using a standard white reference panel before each measurement, with the averaging set to 10 measurements and an integration time of 100ms. Reflectance data were collected at 2,151 wavelength points. For each variety, 150 kernels were randomly selected for spectral measurement, with the spectroradiometer being recalibrated before each measurement to ensure data consistency and accuracy. All measurements were conducted under identical laboratory conditions, with strict environmental control and standardized operational procedures ensuring the reliability and reproducibility of the spectral data.

**Figure 3:**
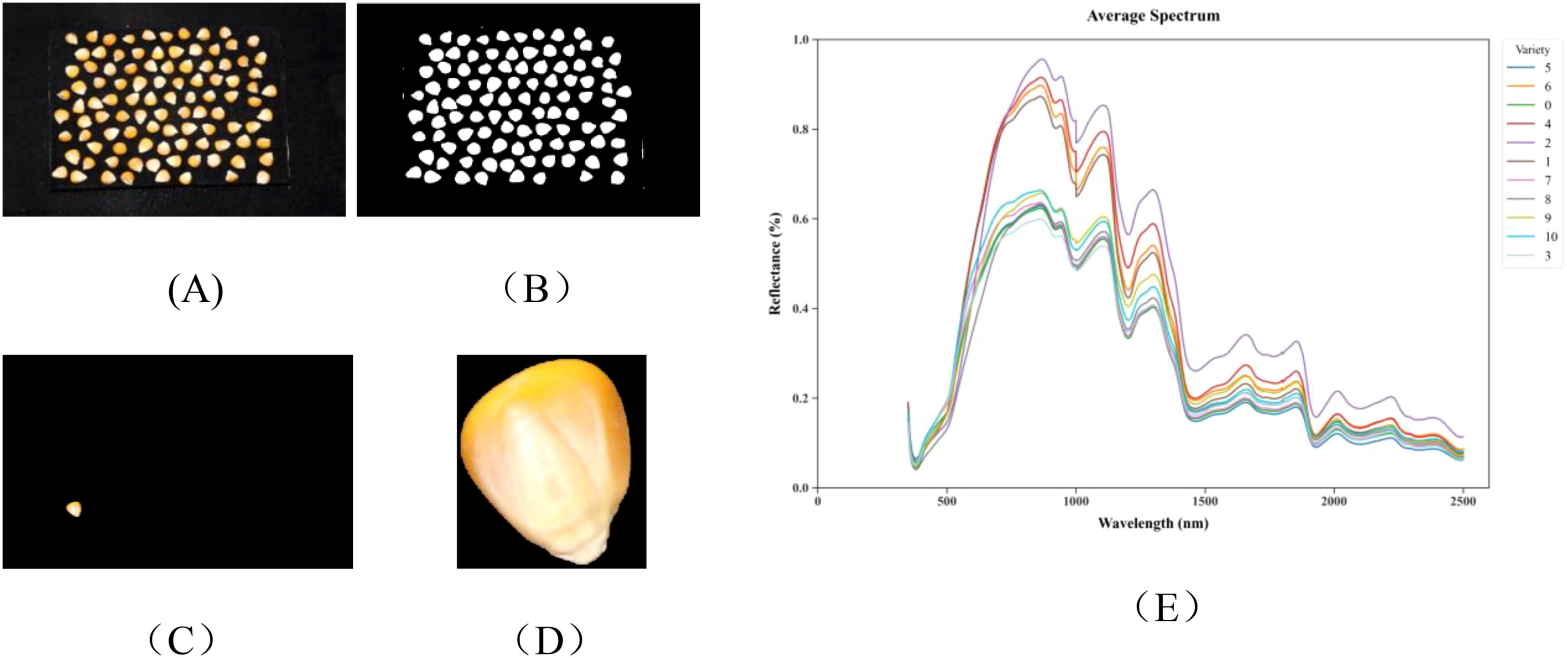
The preprocessed data: **(A)** Image data, original image; **(B)** binarized image; **(C)** mask image; **(D)** segmented image; **(E)** average hyperspectral curves of 11 maize varieties.

The raw RGB images were first converted to grayscale and denoised using Gaussian filtering. Subsequently, Otsu’s method was applied to obtain binary threshold images, followed by morphological opening operations to remove residual noise. Background regions were then identified through dilation operations, while foreground regions were recognized using distance transformation. The unknown regions were determined through subtraction operations. Furthermore, the watershed algorithm was employed for image segmentation of the foreground regions, and a boundary tracking algorithm was used to extract the contours of individual maize kernels. Finally, 100 maize kernels from each image were segmented into individual kernel images to facilitate subsequent feature extraction and analysis. As shown in **Figures 3A-D**.

For hyperspectral data, surface scatter reflection from samples may cause data variations among identical sample types, increasing noise and affecting model accuracy. Therefore, Savitzky-Golay (SG) smoothing technique is employed. This method reduces noise by fitting polynomials within a sliding window, thereby enhancing the signal-to-noise ratio while preserving spectral details, thus improving data correlation and model discrimination precision. Preprocessed data are more suitable for feature extraction and model training. The average spectral curves after preprocessing are shown in **Figure 3E**, demonstrating unique spectral characteristics of 11 maize varieties within the 350–2500 nm wavelength range. The overall trend of spectral curves for all varieties appears similar, with distinct peaks near 863 nm, 1105 nm, 1295 nm, 1680 nm, and 2015 nm, and notable valleys near 980 nm, 1175 nm, 1450 nm, 1780 nm, and 1915 nm. However, significant differences exist in reflection intensity at specific wavebands. These differences are primarily distributed across the following key regions: Visible light region (350–780 nm): Primarily associated with grain pigment characteristics, with 450–550 nm reflecting carotenoid content and 550–680 nm reflecting chlorophyll residues, directly influencing grain color properties. Near-infrared region (780–1100 nm): The absorption valley at 970–980 nm corresponds to O-H bond absorption in water molecules, while 1000–1100 nm relates to C-H bond vibrations in carbohydrates, reflecting grain moisture content and starch properties. Short-wave infrared region (1100–2500 nm): Encompasses multiple key characteristic wavebands, with 1210–1230 nm reflecting lipid C-H bond properties, 1450–1480 nm corresponding to water-related O-H bond absorption, 1500–1570 nm manifesting protein N-H bond characteristics, 2100–2200 nm characterizing starch C-O-H bond absorption, and 2300–2350 nm associated with lipid C-H bonds.

The positional and intensity differences of these characteristic peaks and valleys reflect structural variations in major biochemical components such as proteins, lipids, and carbohydrates among different maize varieties, providing spectral evidence for variety identification. The spectral differences between varieties are most significant in the 1400–1600 nm and 2000–2300 nm intervals, containing absorption characteristics of multiple functional groups (C-H, N-H, and O-H), constituting critical wavelength regions for deep learning models to identify varieties.

### 2.2 Network architecture

#### 2.2.1 Overall structure

**Figure 4:**
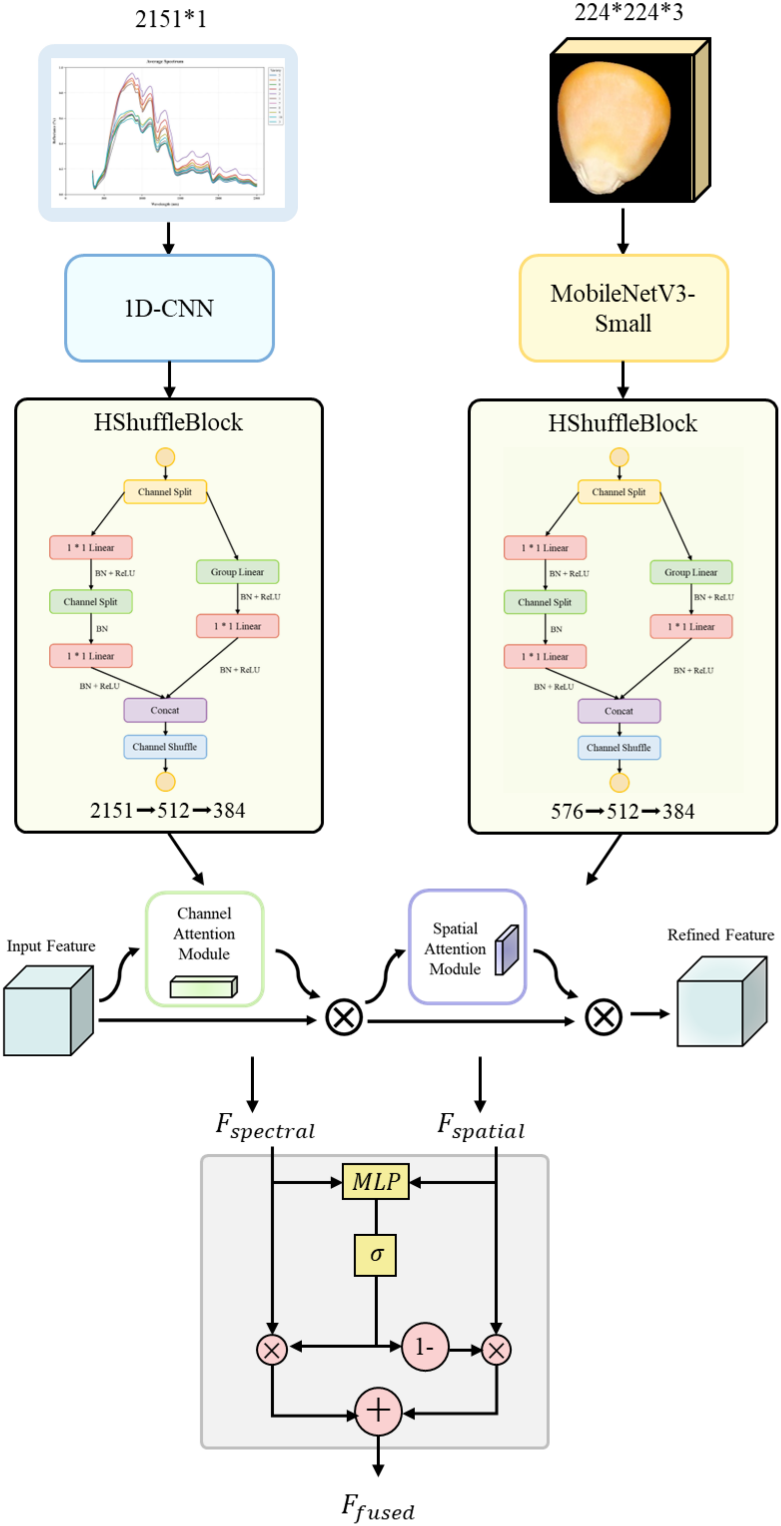
Overall architecture diagram.

The proposed DualCMNet adopts a dual-branch parallel architecture to process hyperspectral data and RGB image data separately. As shown in **Figure 4**, the network comprises four key modules: a dual-branch feature extraction network, an HShuffleBlock feature transformation module, a Convolutional Block Attention Module (CBAM), and a lightweight gated fusion module. Specifically, the dual-branch feature extraction network employs a one-dimensional convolutional network (1D-CNN) and MobileNetV3 to process spectral and spatial data, respectively. The HShuffleBlock achieves feature dimension alignment and information interaction through grouped linear transformation and channel shuffling. The CBAM enhances the representation of discriminative features through channel and spatial attention, thereby improving the model’s ability to recognize subtle differences between varieties. The lightweight gated fusion module dynamically regulates the feature fusion process through adaptive weights. This modular design achieves efficient spectral-spatial feature fusion while maintaining low computational complexity.

#### 2.2.2 Double branch feature extraction

**Figure 5:**
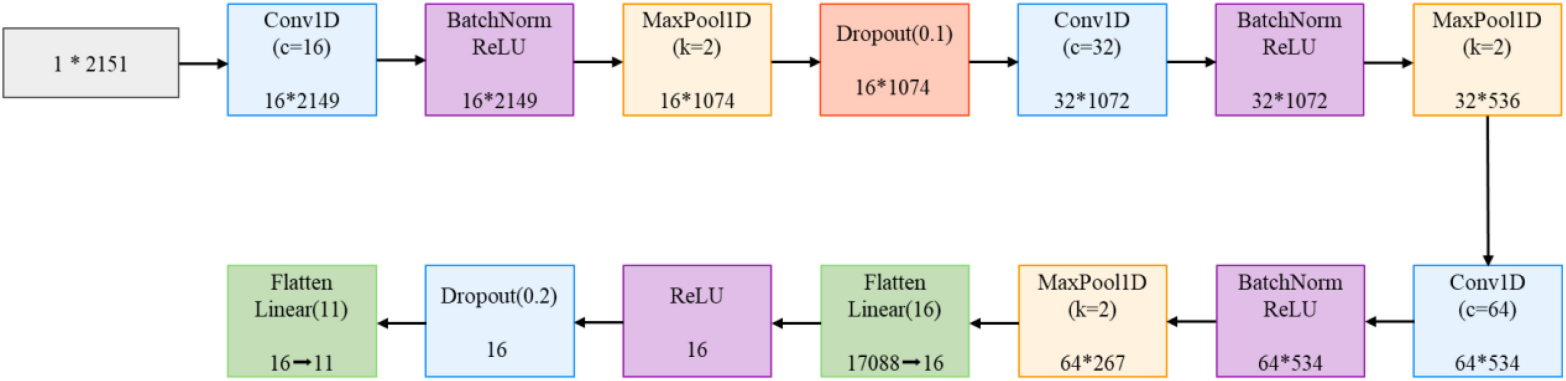
1D-CNN structure.

The feature extraction module adopts a dual-branch architecture, with dedicated feature extraction networks designed for hyperspectral data and RGB images respectively. The spectral branch employs 1D-CNN to process 2151-dimensional hyperspectral data. As shown in **Figure 5**, the network comprises three consecutive convolution blocks with channel dimensions of 16, 32, and 64, respectively. Each convolution block consists of one-dimensional convolution, batch normalization, ReLU activation function, and max-pooling layer, with the first convolution block additionally incorporating a Dropout layer. This progressive feature extraction strategy enables the network to effectively capture spectral features from local to global scales. The flattened features are ultimately mapped through two fully connected layers and a Dropout layer.

**Figure 6:**
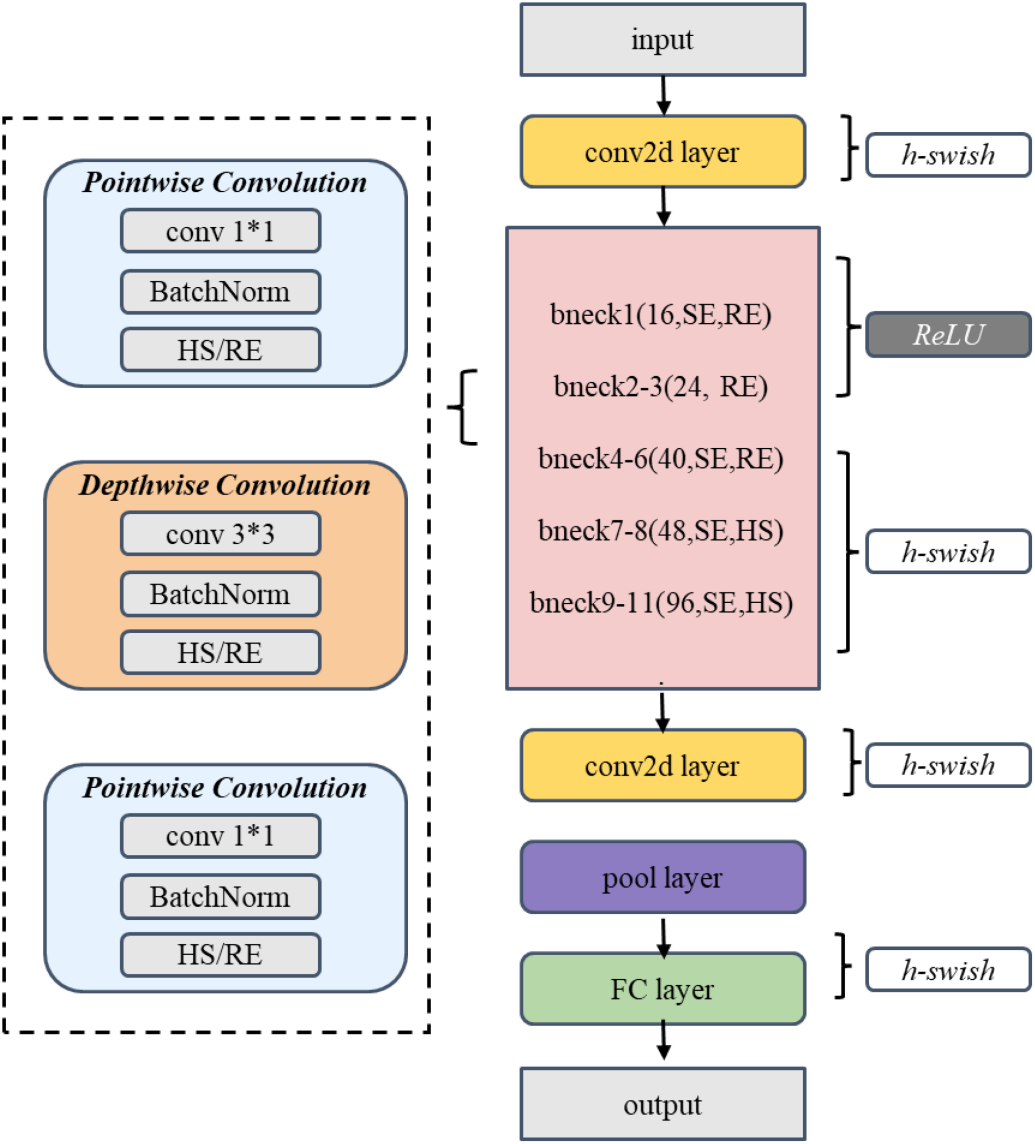
MobileNetV3-Small Structure diagram.

The spatial branch employs MobileNetV3-Small as the feature extraction network (Howard et al., 2019). The Small version, rather than the Large version, was selected primarily due to its lower computational overhead and memory footprint, taking into consideration the resource constraints in practical deployment scenarios. As illustrated in **Figure 6** and **Table 1**, the network extracts initial features through a 3×3 convolutional layer, followed by eleven inverted residual blocks that progressively construct feature representations. To enhance feature discriminative capability, the network incorporates SE attention mechanisms at specific layers and flexibly configures Hardswish/ReLU activation functions. Finally, a 1024-dimensional feature vector is output through adaptive average pooling and fully connected layers.

**Table 1:** MobileNetV3-Small Architecture.

Input Size	Operation	Elevated Dimension	Output Channel	SE	Activation Function	Padding
224 × 224 × 3	Conv2d, 3 × 3	–	16	–	H-Swish	2
112 × 112 × 16	Bottleneck, 3 × 3	16	16	✓	Relu6	2
56 × 56 × 16	Bottleneck, 3 × 3	72	24	–	Relu6	2
28 × 28 × 24	Bottleneck, 3 × 3	88	24	–	Relu6	1
28 × 28 × 24	Bottleneck, 5 × 5	96	40	✓	H-Swish	2
14 × 14 × 40	Bottleneck, 5 × 5	240	40	✓	H-Swish	1
14 × 14 × 40	Bottleneck, 5 × 5	240	40	✓	H-Swish	1
14 × 14 × 40	Bottleneck, 5 × 5	120	48	✓	H-Swish	1
14 × 14 × 48	Bottleneck, 5 × 5	144	48	✓	H-Swish	1
14 × 14 × 48	Bottleneck, 5 × 5	288	96	✓	H-Swish	2
7 × 7 × 96	Bottleneck, 5 × 5	576	96	✓	H-Swish	1
7 × 7 × 96	Bottleneck, 5 × 5	576	96	✓	H-Swish	1
7 × 7 × 96	Conv2d, 1 × 1	–	576	✓	H-Swish	1
7 × 7 × 576	Pool, 7 × 7	–	–	–	–	1
1 × 1 × 576	Fc, 1 × 1	–	1024	–	H-Swish	1
1 × 1 × 1024	Fc, 1 × 1	–	k	–	–	1

√ indicates the module is included in the network architecture, while blank cells indicate the module is not used.

#### 2.2.3 Feature transformation module

**Figure 7:**
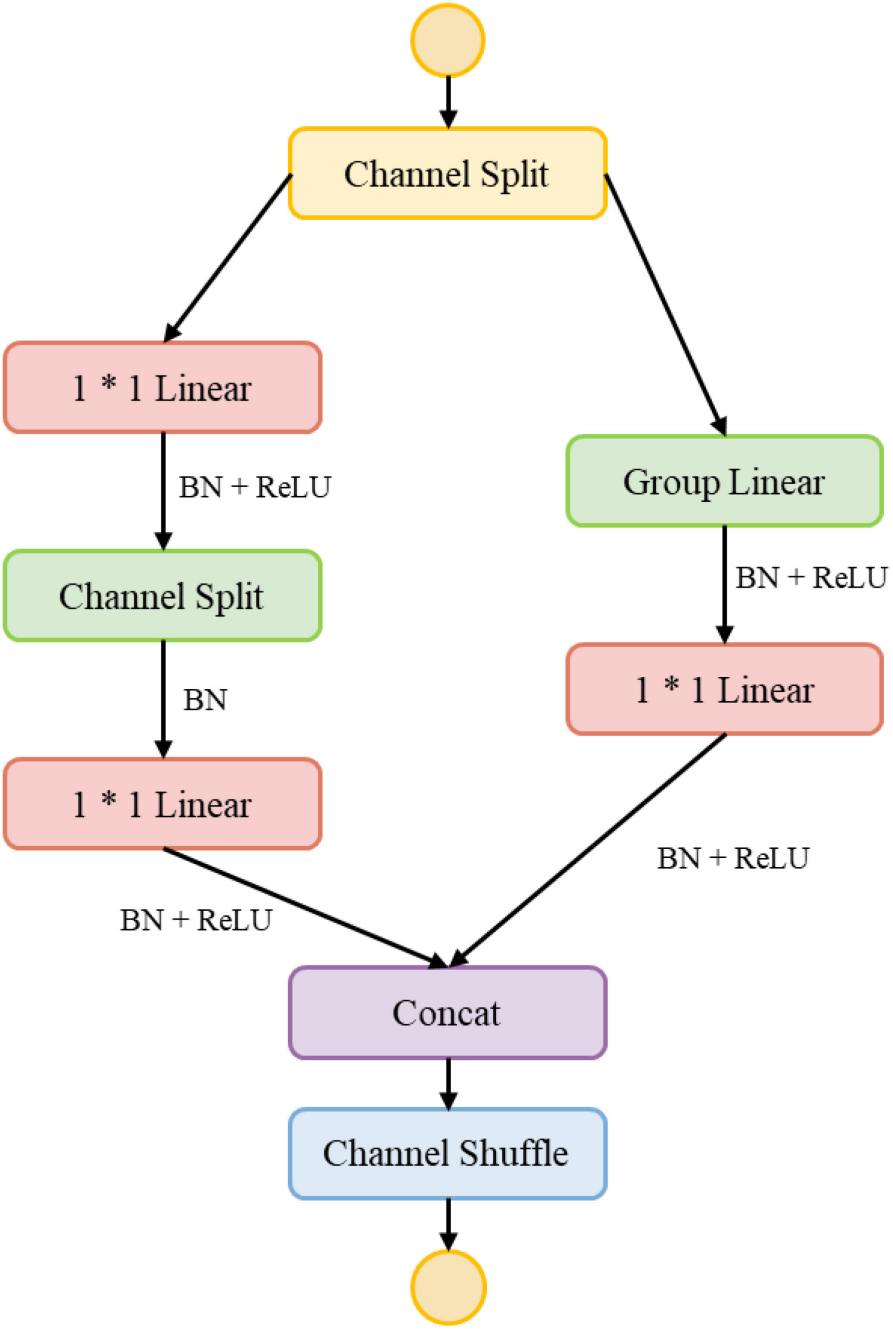
HShuffleBlock basic structure.

To achieve efficient fusion of multi-modal features, this study designs a feature transformation module HShuffleBlock based on ShuffleBlock (Ma et al., 2018). Through grouped linear transformation and channel shuffling operations, this module effectively reduces computational complexity while maintaining feature expressiveness. The basic structure of the feature transformation module is shown in **Figure 7**.

The module consists of two main components: grouped linear transformation and channel shuffling. The grouped linear transformation first divides the input feature $X\in {\mathbb{R}}^{d}$ into *g* groups(*g*=8 in this study), with each group undergoing independent feature transformation. For the i th group feature $X_{i}$ , the transformation process is shown in Equation 1.


(1) $$\begin{array}{c} X_{i}^{transformed}=W_{i}X_{i}+b_{i} \end{array}$$

where $W_{i}$ is the weight matrix of group *i* and $b_{i}$ is the bias term. This grouped transformation strategy reduces the computational complexity from $O(d_{1}d_{2})$ to $O(\frac{d_{1}d_{2}}{g})$ , where $d_{1}$ and $d_{2}$ represent the input and output dimensions, respectively.

The channel shuffling operation achieves inter-group information exchange through feature channel reordering. Given the transformed feature $Y\in {\mathbb{R}}^{d^{\prime }}$ , the channel shuffling process is shown in Equation 2.


(2) $$\begin{array}{c} \begin{array}{c} Y_{reshaped}=reshape(Y,(batch,g,\frac{d^{\prime }}{g}))\\ Y_{shuffled}=transpose(Y_{reshaped},(0,2,1))\\ Y_{output}=reshape(Y_{shuffled},(batch,d^{\prime })) \end{array} \end{array}$$

To enhance feature expressiveness, the module adopts a two-stage structure, with its overall transformation process is shown in Equation 3.


(3) $$\begin{array}{c} \begin{array}{c} F_{mid}=\sigma (\text{BN}({\text{ShuffleBlock}}_{1}(X)))\\ F_{out}=\sigma (\text{BN}({\text{ShuffleBlock}}_{2}(F_{mid}))) \end{array} \end{array}$$

where σ denotes the ReLU activation function and BN represents batch normalization. The first ShuffleBlock maps features to a 512-dimensional intermediate feature space, while the second maps features further to a 384-dimensional common feature space, as shown in Equation 4.


(4) $$\begin{array}{c} X\in {\mathbb{R}}^{d}\overset{{\text{ShuffleBlock}}_{1}}{\to }{\mathbb{R}}^{512}\overset{{\text{ShuffleBlock}}_{2}}{\to }{\mathbb{R}}^{384} \end{array}$$

After processing through the feature transformation module, both spectral features $F_{s}$ and $F_{r}$ are mapped to a feature space of identical dimensionality, as shown in Equation 5.


(5) $$\begin{array}{c} F_{s}^{transformed},F_{r}^{transformed}\in {\mathbb{R}}^{384} \end{array}$$

The design of the feature transformation module effectively addresses the dimensional mismatch problem in multi-modal feature fusion while significantly reducing computational overhead, thereby providing a solid foundation for subsequent attention enhancement and feature fusion.

#### 2.2.4 Attention-enhancing mechanisms

In this study, Convolutional Block Attention Module(CBAM) attention mechanism (Woo et al., 2018) s used to enhance feature expression. In the proposed network, CBAM acts on the transformed spectral and spatial features respectively, and enhances the discriminative ability of the features through the series-connected channel and spatial attention modules.

**Figure 8:**
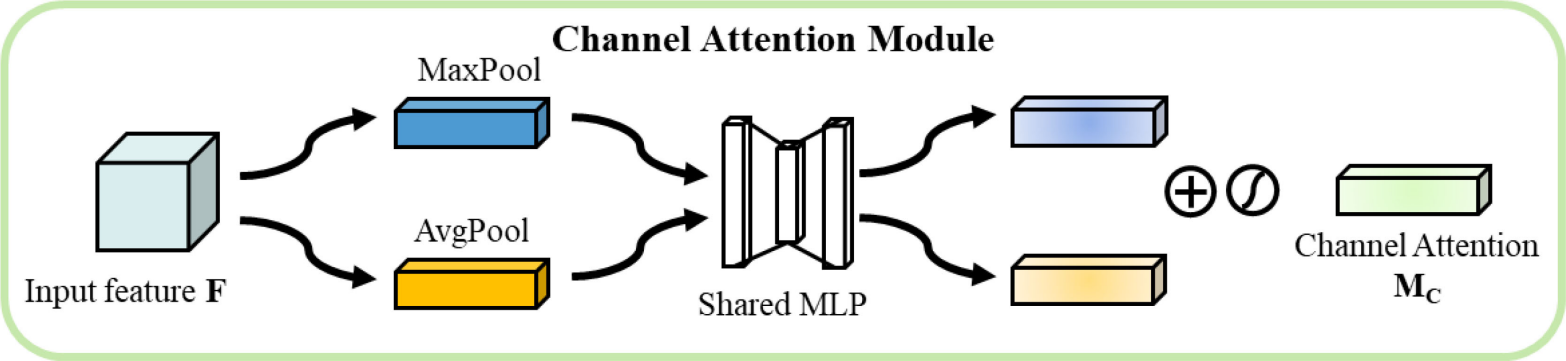
Channel attention.


The channel attention module extracts channel statistics through parallel adaptive average pooling and maximum pooling operations As shown in **Figure 8**. For input feature $F\in {\mathbb{R}}^{C\times H\times W}$ , the average and maximum values along the channel dimension are calculated as shown in Equation 6.

(6) $$\begin{array}{c} \begin{array}{c} Z_{\text{avg}}=\text{AvgPool}(F)\in {\mathbb{R}}^{C}\\ Z_{\text{max}}=\text{MaxPool}(F)\in {\mathbb{R}}^{C} \end{array} \end{array}$$

These features undergo nonlinear transformation through a shared-weight multilayer perceptron as shown in Equation 7.


(7) $$\begin{array}{c} MLP(Z)=W_{2}\cdot ReLU(W_{1}\cdot Z) \end{array}$$

where $W_{1}\in {\mathbb{R}}^{\frac{C}{r}\times C}$ and $W_{2}\in {\mathbb{R}}^{C\times \frac{C}{r}}$ are the learnable weight matrices and *r* is the downscaling ratio. The channel attention weights are computed by the following, as shown in Equation 8.


(8) $$\begin{array}{c} M_{c}(F)=\sigma (\text{MLP}(Z_{\text{avg}})+\text{MLP}(Z_{\text{max}})) \end{array}$$

where σ is the sigmoid activation function.

**Figure 9:**
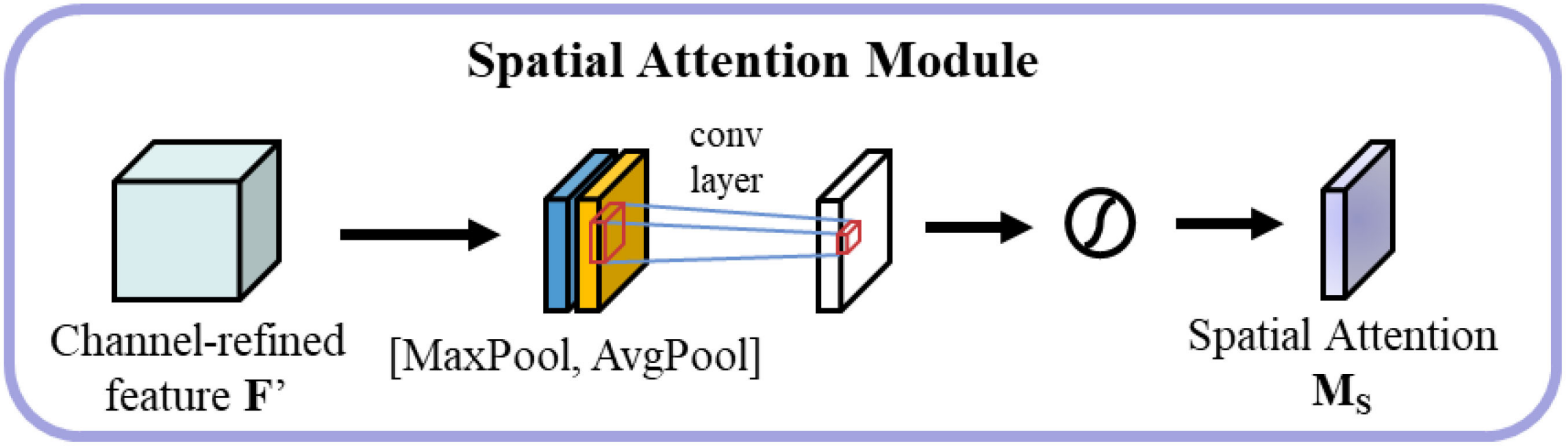
Spatial Attention.


The spatial attention module focuses on the spatial distribution of features As shown in **Figure 9**. Initially, average pooling and maximum pooling are performed along the channel dimension, followed by concatenation and processing through a 7×7 convolutional layer to generate spatial attention weights, as shown in Equation 9.

(9) $$\begin{array}{c} \begin{array}{c} S_{\text{avg}}={\text{AvgPool}}_{\text{spatial}}(F)\in {\mathbb{R}}^{H\times W}\\ S_{\text{max}}={\text{MaxPool}}_{\text{spatial}}(F)\in {\mathbb{R}}^{H\times W}\\ M_{s}(F)=\sigma ({\text{Conv}}_{7\times 7}([S_{\text{avg}};S_{\text{max}}])) \end{array} \end{array}$$

where $\left[S_{\text{avg}};S_{\text{max}}\right]$ represents the concatenation operation, and σ is the sigmoid activation function.

The sequential application of channel and spatial attention proceeds as follows, as shown in Equation 10.


(10) $$\begin{array}{c} \begin{array}{c} F_{1}=M_{c}(F)⊗F\\ F_{\text{out}}=M_{s}(F_{1})⊗F_{1} \end{array} \end{array}$$

where ⊗ denotes element-by-element multiplication. For spectral features, channel attention emphasizes the contribution of important wavelength bands; for spatial features, spatial attention highlights critical regions of grain morphology.

#### 2.2.5 Feature fusion strategy

**Figure 10:**
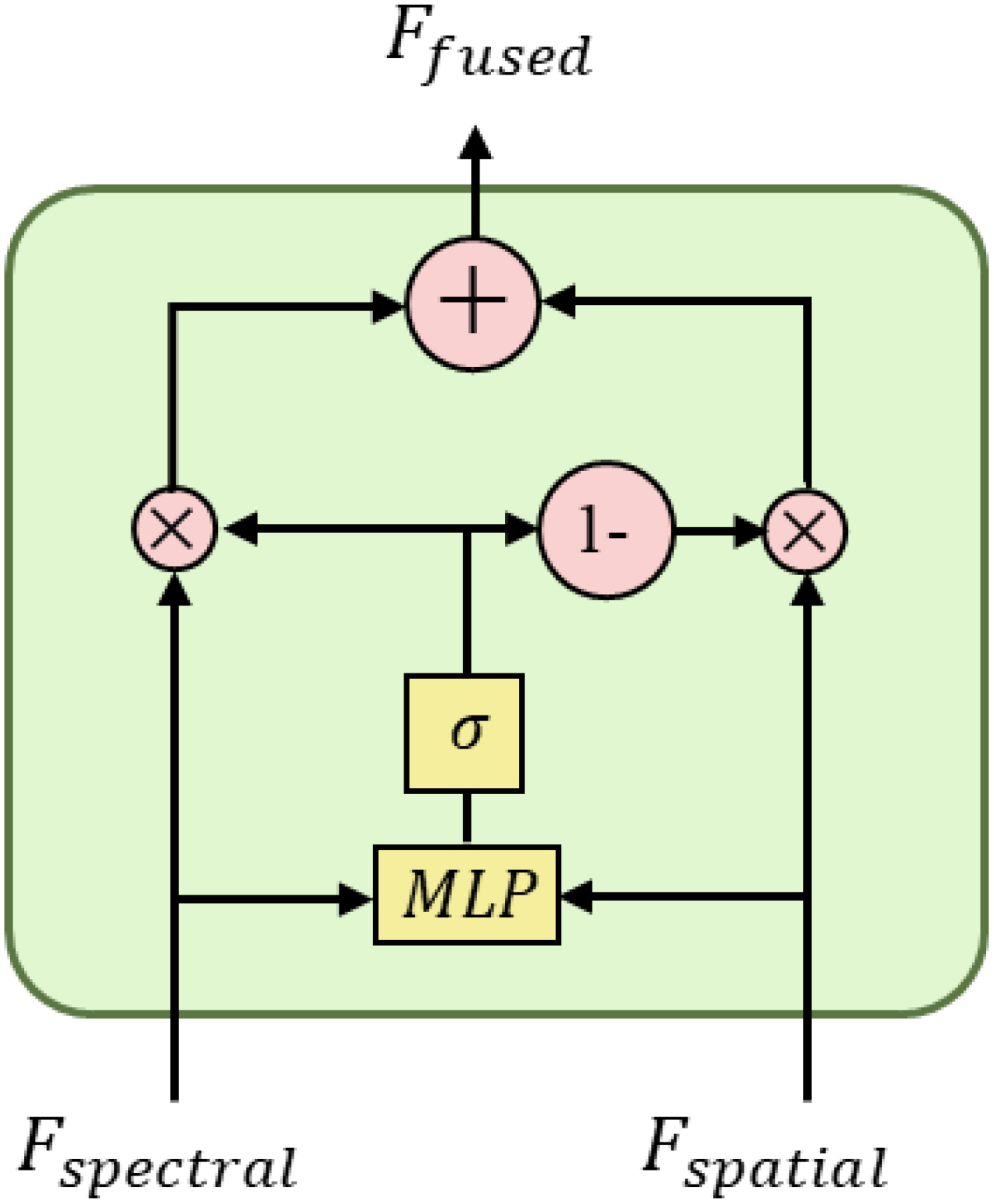
Lightweight gating mechanism.

To effectively integrate the complementary information of spectral and spatial features, this study designed a lightweight gated fusion module. This module dynamically adjusts the contribution of different modal features by learning adaptive gating values while balancing computational efficiency and fusion performance (Arevalo et al., 2017). The structure of the gated fusion module is shown in **Figure 10**.

The gated network adopts a simplified two-layer perceptron structure with an input dimension of 2×384 (concatenated bimodal features), a middle layer dimension of 48, and an output dimension of 1. In order to improve training stability and generalization capability, ReLU activation function and dropout layer (dropout rate 0.2) are inserted between the two fully connected layers. The gated network design follows the “narrow-wide” lightweight principle, and significantly reducing the number of parameters by decreasing the middle layer dimension.

The fusion strategy considers both multiplicative interaction and additive combination of features, which can be mathematically expressed as shown in Equation 11.


(11) $$\begin{array}{c} \begin{array}{c} g=\sigma (\text{MLP}([F_{\text{spectral}},F_{\text{spatial}}]))\\ F_{\text{fused}}=g\cdot (F_{\text{spectral}}*F_{\text{spatial}})+(1-g)\cdot (F_{\text{spectral}}+F_{\text{spatial}}) \end{array} \end{array}$$

where *g* represents the fusion weight output by the gating network, ranging from [0,1]; σ denotes the sigmoid activation function; [·,·] represents the feature concatenation operation; $F_{\text{spectral}}$ and $F_{\text{spatial}}$ represent the spectral and spatial features after feature transformation and attention enhancement, respectively.

The fusion strategy captures the nonlinear correlation between modalities through the multiplicative interaction term, which helps to discover the composite features for maize seed variety discrimination, and at the same time utilizes the additive combination term to retain the independent information of each modality, preventing the effective features from being lost in the fusion process. The adaptive gating mechanism can dynamically adjust the weights of the two fusion modes according to the characteristics of the input data to improve the adaptability and robustness of the model. Compared with simple feature splicing or weighted averaging methods, this fusion strategy can better integrate discriminative information in the spectral and spatial domains.

### 2.3 Physicochemical and morphological characteristics of maize varieties

Morphological characteristics and physiological properties serve as crucial identifiers for maize varieties and form the foundation for multimodal data fusion. This study conducted a systematic analysis of grain morphological features and physicochemical properties reflected by spectral response across 11 maize varieties, providing support for a deeper understanding of the recognition mechanism in the proposed DualCMNet model.

#### 2.3.1 Analysis of grain morphological characteristics

Grain morphological characteristics are visually observable physical properties, primarily manifested in aspects such as size, shape, and color. **Table 2** presents the main morphological characteristics of the 11 maize varieties used in this study.

**Table 2:** Main morphological characteristics of 11 maize varieties.

Variety	Average Length (mm)	Average Width (mm)	Thousand-grain Weight (g)	Color	Type
JiDan436	11.8 ± 0.6	8.0 ± 0.4	367	Yellow	dent
JiDan50	12.0 ± 0.6	7.5 ± 0.4	300	Yellow	Semi-dent
JiDan505	12.3 ± 0.6	8.3 ± 0.4	330	Orange	Dent
JiDan83	11.0 ± 0.5	8.8 ± 0.4	364	Yellow	Semi-dent
JiDan209	10.5 ± 0.5	9.0 ± 0.4	380	Yellow	Semi-dent
JiDan407	11.5 ± 0.6	8.2 ± 0.4	370	Yellow	Dent
JiDan27	10.8 ± 0.5	9.2 ± 0.4	400	Yellow	Semi-dent
JiDan626	11.2 ± 0.5	8.6 ± 0.4	416	Yellow	Semi-dent
JiDan953	11.0 ± 0.5	8.8 ± 0.4	372	Yellow	Dent
ZD958	10.2 ± 0.5	8.0 ± 0.4	345	Yellow	Semi-dent
LY9915	12.2 ± 0.6	8.5 ± 0.4	385	Orange	Dent

Based on morphological characteristic analysis, these varieties can be categorized into two main types: (1) Dent maize, including JiDan436, JiDan505, JiDan407, JiDan953, and LY9915, characterized by larger length-to-width ratios and more elongated elliptical shapes; (2) Semi-dent maize, including JiDan50, JiDan83, JiDan209, JiDan27, JiDan626, and ZD958, exhibiting greater variation in length-to-width ratios. Notably, JiDan27 and JiDan209 demonstrated the lowest length-to-width ratios (both 1.17), presenting a more rounded appearance, while JiDan50, despite being classified as semi-dent, exhibited a length-to-width ratio (1.60) higher than all dent varieties, displaying a uniquely slender morphology. Regarding color characteristics, most varieties appeared yellow, whereas JiDan505 and LY9915 presented orange coloration, providing important visual cues for image-based identification. Additionally, significant differences were observed in thousand-kernel weight, ranging from the lowest value in JiDan50 (300g) to the highest in JiDan626 (416g); this physical property, closely related to kernel volume and density, serves as a supplementary feature for varietal identification.

#### 2.3.2 Spectral characteristics and physicochemical properties

Hyperspectral data reflects the internal physicochemical composition of grains, including the relative content of components such as starch, protein, and fat. In this study, the differences in crude protein content among maize varieties ranged from 8.46% (JiDan27) to 10.92% (JiDan83), crude fat content varied from 3.23% (JiDan407) to 4.99% (LY9915), and crude starch content ranged from 68.50% (JiDan209) to 77.33% (JiDan953). These variations in component content constitute the primary cause of spectral differences.

Comparing the data in **Table 3** with the spectral curves in **Figure 3E**, it is evident that varieties with similar physicochemical component contents typically exhibit similar spectral characteristics. For instance, JiDan953 and JiDan407 have comparable crude starch contents (77.33% and 76.60% respectively), and their spectral features in the near-infrared region are relatively similar. Conversely, JiDan209, with a significantly lower crude starch content (68.50%) than other varieties, displays notable differences in spectral characteristics in the corresponding bands. Furthermore, systematic differences in physicochemical composition exist between dent and semi-dent varieties. Comparison of data in **Tables 2**, **3** indicates that dent varieties (such as JiDan436, JiDan407, and JiDan953) typically contain higher crude starch content, averaging 76.77%, while semi-dent varieties (such as JiDan83, JiDan50, and JiDan209) have an average crude starch content of 71.57%. This systematic difference is also reflected in their spectral characteristics.

**Table 3:** Physicochemical indicators of 11 maize varieties.

Variety	Crude Protein Content (%)	Crude Fat Content (%)	Crude Starch Content (%)
JiDan436	10.65	3.57	76.39
JiDan50	9.51	4.31	72.6
JiDan505	9.59	4.7	73.27
JiDan83	10.92	3.66	73.62
JiDan209	10.02	4.55	68.5
JiDan407	10.03	3.23	76.6
JiDan27	8.46	4.06	75.23
JiDan626	8.66	3.99	75.62
JiDan953	8.81	3.67	77.33
ZD958	8.47	3.92	73.42
LY9915	10.58	4.99	73.3

### 2.4 Experimental design

#### 2.4.1 Data segmentation and training strategies

This study employs a two-stage training strategy, comprising pretraining and fusion network training phases. During the pretraining phase, two subnetworks are trained separately using the data collected as described in Section 2.1.2. The spectral branch utilizes one-dimensional convolutional neural networks for feature extraction based on the collected hyperspectral data, while the spatial branch adopts the MobileNetV3-Small architecture for feature learning from RGB images. Both branches are trained for 200 epochs until convergence. In the fusion network training phase, a category-matching data fusion strategy is designed to address the sample alignment issue between the two modalities. Initially, hyperspectral data is organized by variety categories to establish category-feature mapping relationships. For each RGB image sample, a feature vector is randomly selected from the hyperspectral feature set of the same category for matching, forming paired training samples. This category-based random matching strategy ensures category consistency between different modalities while enhancing data diversity through random selection.

Five-fold cross-validation is employed to evaluate model performance. Based on stratified sampling principles, the StratifiedKFold method is used to divide the paired dataset into 5 subsets, ensuring consistent variety sample proportions in each subset. In each fold validation, four subsets (800 pairs per variety, totaling 8,800 paired samples) are randomly selected as the training set, while the remaining subset (200 pairs per variety, totaling 2,200 paired samples) serves as the validation set. RGB images undergo standard preprocessing, with random cropping and random horizontal flipping applied to the training set, and center cropping to the validation set, followed by normalization. Hyperspectral data maintains its original feature vector form to ensure the integrity of spectral information.

The model training adopts a hierarchical optimization strategy with a batch size of 32. An Adam optimizer is employed with a base learning rate of 1×10^-3 and a weight decay coefficient of 1×10^-4. During the 14th epochs, the pretrained feature extraction layers are frozen, with the learning rate for pretrained layers set to 0.1 times the base learning rate. After the 15th epoch, all layers are unfrozen, with the learning rate for pretrained layers adjusted to 0.05 times the base learning rate, and the fusion layer learning rate reduced to 0.1 times the base learning rate. The training process continues for 50 epochs using a cross-entropy loss function. Model performance is evaluated on the validation set after each epoch, and the model parameters with the highest validation accuracy are saved.

#### 2.4.2 Evaluation indicators

This research employs multiple metrics to evaluate model performance, including Accuracy, Precision, Recall, F1 score, Parameters, FLOPs, and Confusion Matrix. For a dataset D with n samples, these metrics are calculated as follows:


$$\text{Accuracy}=\frac{\sum_{i=1}^{n}I\left(y_{i}={\hat{y}}_{i}\right)}{n}$$

where $y_{i}$ represents the true label of the i th sample, ${\hat{y}}_{i}$ denotes the predicted label of the i th  sample, and I(·) is an indicator function that returns 1 when $y_{i}={\hat{y}}_{i}$ and 0 otherwise.

Precision and Recall are defined as:


$$\text{Precision}=\frac{TP}{TP+FP},\text{Recall}=\frac{TP}{TP+FN}$$

where *TP*(True Positive) represents the number of samples correctly predicted as positive, *FP*(False Positive) indicates the number of samples incorrectly predicted as positive, and *FN* (False Negative) denotes the number of samples incorrectly predicted as negative.

The F1 score is calculated as:


$$F1=2\times \frac{\text{Precision}\times \text{Recall}}{\text{Precision}+\text{Recall}}$$

The Confusion Matrix $M\in {\mathbb{R}}^{c\times c}$ represents the model’s prediction performance in multi-classification tasks, where $M_{ij}$ indicates the number of samples from class i that are predicted as class *j*. Additionally, FLOPs and parameter count are used to evaluate the model’s computational complexity. FLOPs represents the total number of floating-point operations executed during one forward inference process, measuring the model’s computational complexity. The parameter count refers to the sum of all trainable parameters in the model, including those in convolutional layers, fully connected layers, and other components, which measures the model’s storage and computational overhead.

## 3 Results

### 3.1 Baseline experiment results and comparison

#### 3.1.1 Comparison of single-modal performance

**Figure 11:**
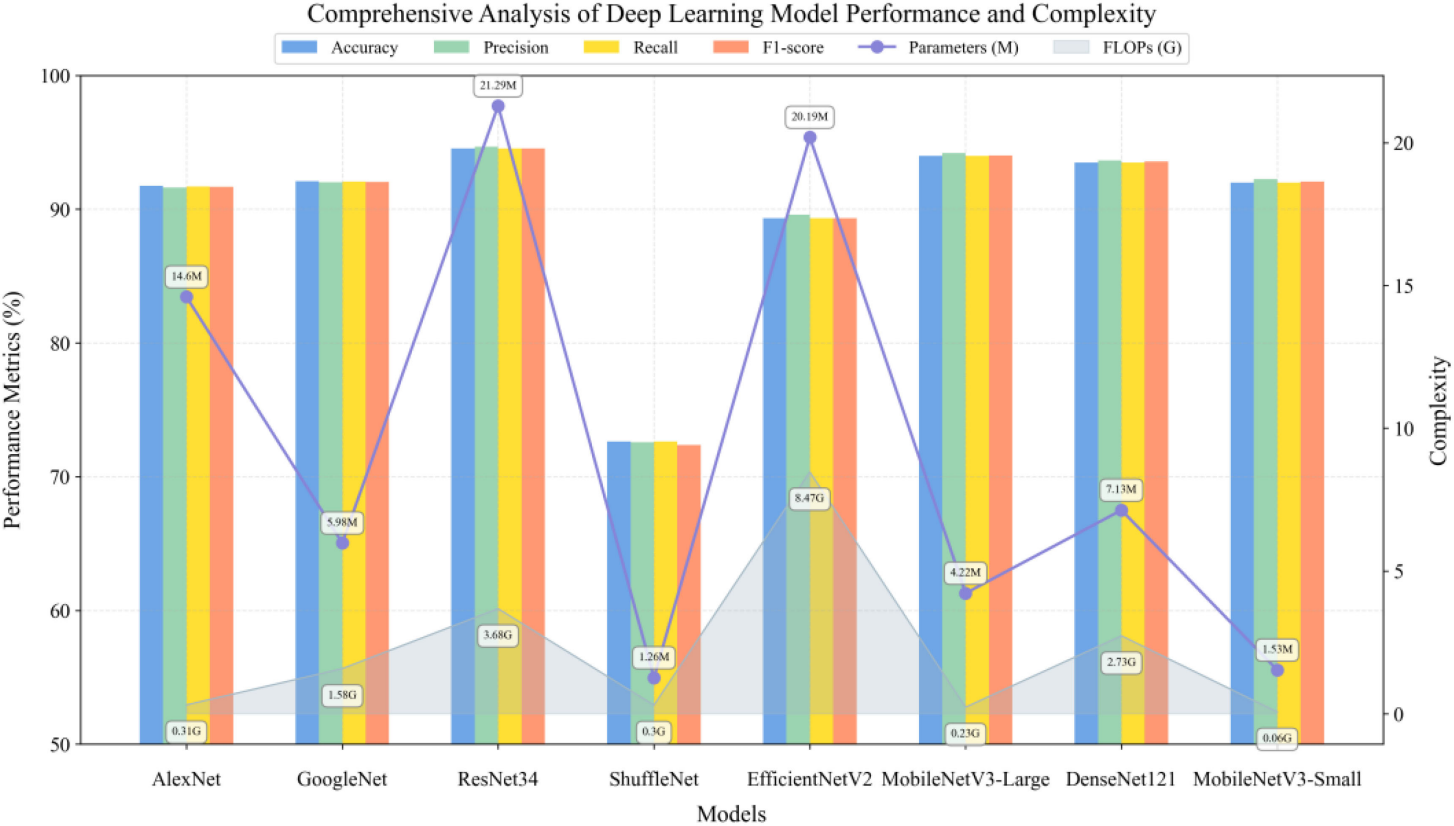
RGB image classification performance.

To evaluate the performance characteristics of deep learning architectures on unimodal data, this study conducted comparative experiments on typical convolutional neural networks for RGB image classification tasks. As shown in **Figure 11**, the experimental results demonstrate that ResNet34 (He et al., 2016) achieved optimal classification performance of 94.54% through its deep residual learning mechanism, albeit with a relatively large parameter scale. Considering this research aims to develop lightweight solutions applicable to practical agricultural scenarios, the MobileNetV3 series, which employs depth-wise separable convolutions and SE attention mechanisms, showed remarkable performance. Specifically, the Large version achieved 94.00% accuracy through hard swish search with only 4.22M parameters, while the Small version further reduces parameters to 1.53M through channel compression, requiring only 0.06 GFLOPs of computational resources while maintaining 92.00% classification accuracy. This exhibits an excellent performance-to-efficiency ratio suitable for resource-constrained agricultural deployment environments. Although DenseNet121 (Huang et al., 2017), based on dense connection structures, achieves 93.50% classification accuracy, its parameter size of 7.13M and computational requirements of 2.73 GFLOPs are significantly higher compared to MobileNetV3-Small, making it difficult to meet lightweight deployment requirements. Notably, although ShuffleNet (Zhang et al., 2018) adopted a lightweight design based on channel shuffling, its accuracy was significantly lower than other models, indicating that excessive lightweighting may compromise feature extraction capabilities. EfficientNetV2 (Tan and Le, 2021), which employs compound scaling strategies, achieved only 89.32% classification accuracy at a larger model scale, demonstrating higher computational costs but lower classification performance compared to the MobileNetV3 series. Among classical architectures, AlexNet (Krizhevsky et al., 2012) with ReLU activation and GoogleNet (Szegedy et al., 2015) based on Inception modules achieved 91.74% and 92.09% accuracy respectively, but their parameter efficiency showed notable gaps compared to modern architectures.

**Figure 12:**
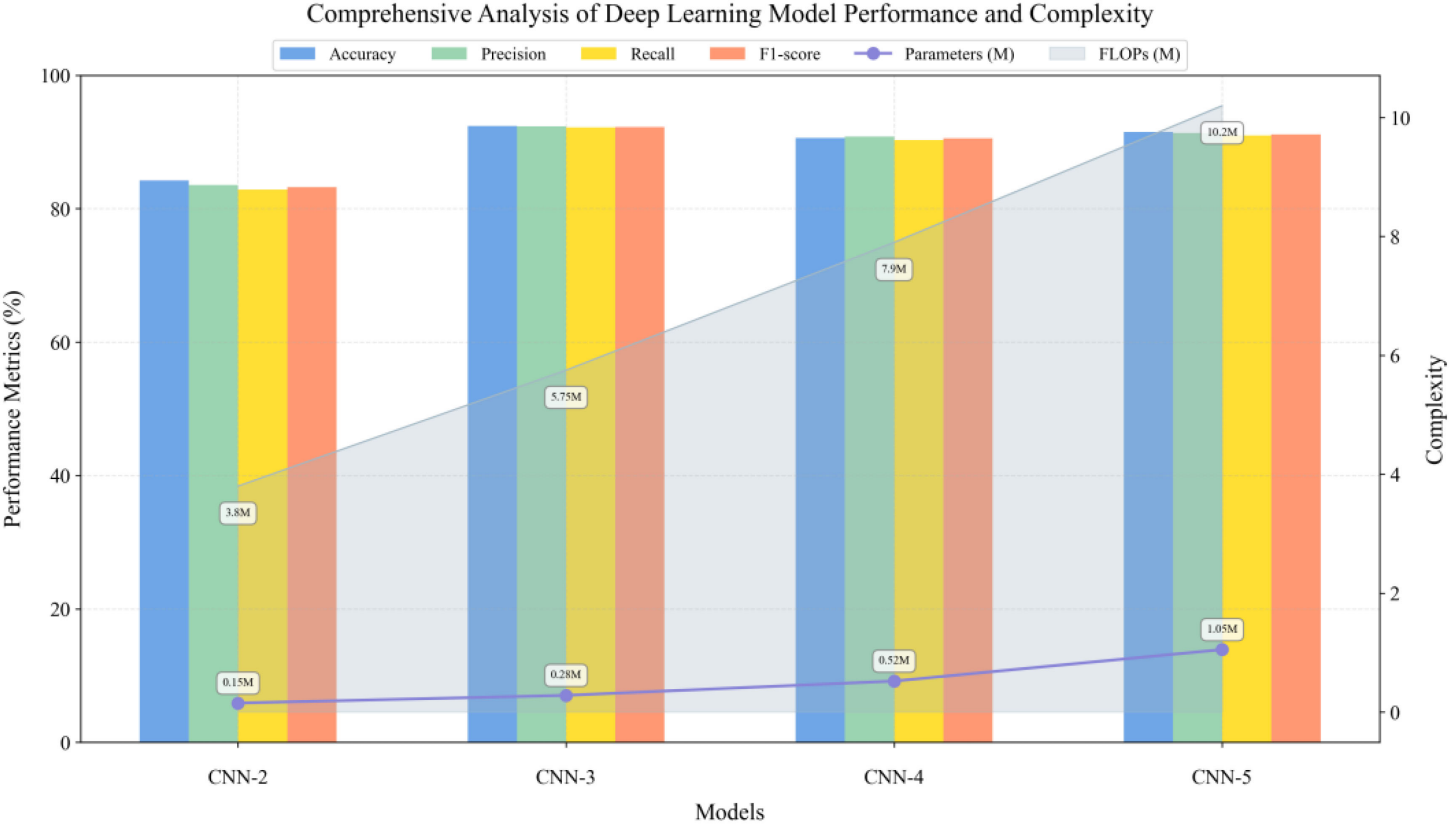
Hyperspectral classification performance.

Meanwhile, this study constructed one-dimensional convolutional neural networks of varying depths to evaluate the impact of network architecture on hyperspectral data classification (**Figure 12**). For each network structure, full-spectrum (350–2500 nm, 2151 wavelength points) spectral data were used as input. The experimental results demonstrated that a three-layer convolutional structure achieved optimal classification performance by progressively increasing feature channels and incorporating batch normalization, max-pooling downsampling, and ReLU activation functions in each convolutional block. This architecture achieved a classification accuracy of 92.42% while requiring only 0.28M parameters and 5.75M FLOPs computational cost. Although CNN-2 employed a larger initial channel number, its classification performance was limited by network depth. When further increasing the network depth to four and five layers, despite attempting to enhance network expressiveness through deeper feature mapping, model performance decreased rather than improved: CNN-4 and CNN-5 achieved accuracies of 90.61% and 91.52%, respectively, while model complexity increased significantly. CNN-5’s parameter count and computational cost were 3.75 times and 1.77 times that of CNN-3, respectively. These experimental results indicate that for hyperspectral data classification tasks, the three-layer convolutional structure achieved an optimal balance between model expressiveness and computational efficiency.

#### 3.1.2 Comparison of multimodal fusion methods

**Figure 13:**
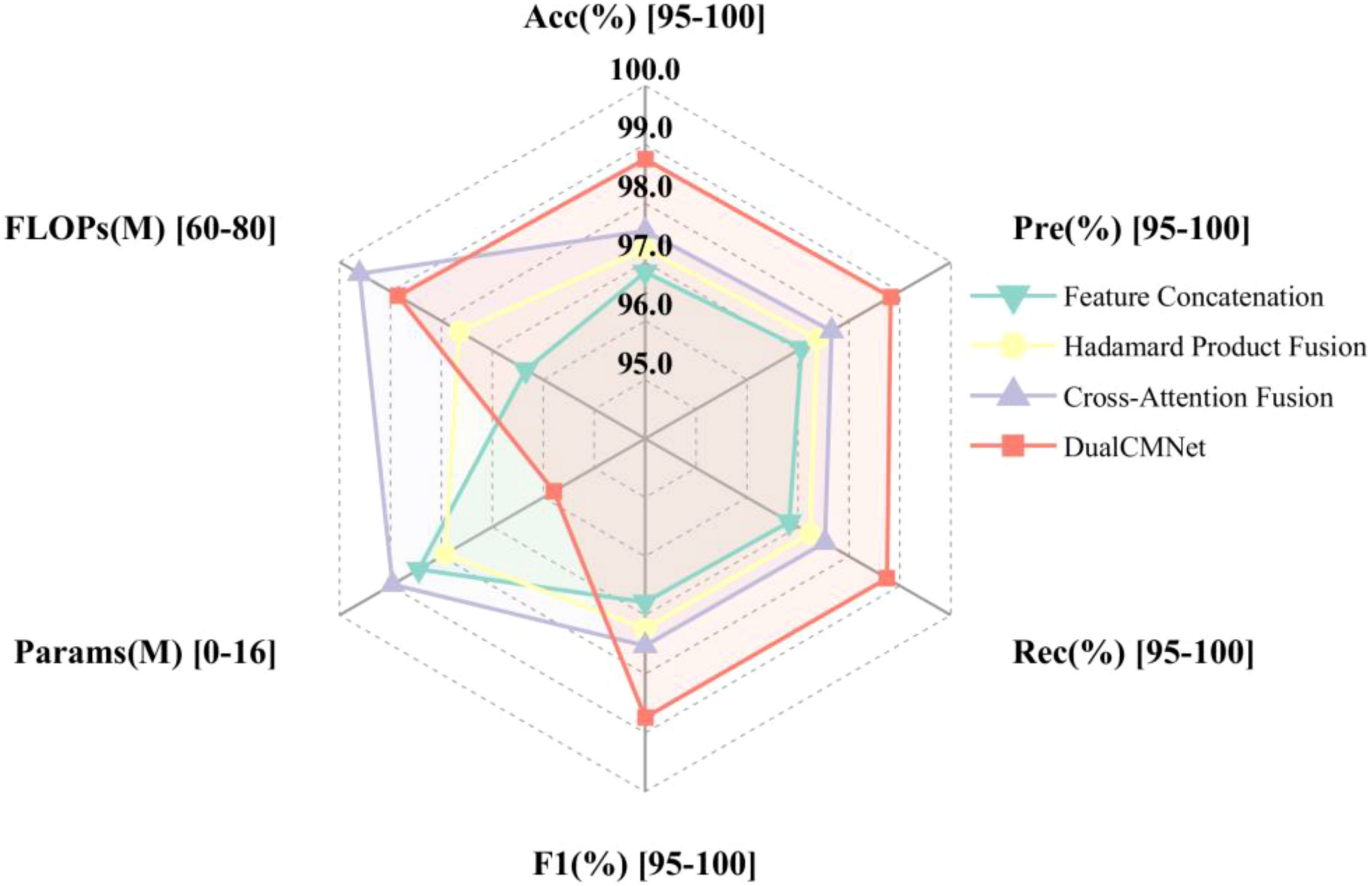
Performance comparison of multimodal fusion methods.

Based on single-modal experimental analysis results, this study selected MobileNetV3-Small (accuracy 92.00%, parameter count 1.53M) and CNN-3 (accuracy 92.42%, parameter count 0.28M), which demonstrated optimal performance and lightweight structures, to construct the spatial feature extraction branch and spectral feature extraction branches, respectively. The effectiveness of different feature fusion strategies was systematically evaluated. **Figure 13** compares the performance metrics of four different fusion methods on the validation set. The experimental results demonstrate that the basic feature concatenation strategy achieved a classification accuracy of 96.83%, validating the effectiveness of spatial-spectral feature fusion. The residual Hadamard product fusion further improved the accuracy to 97.23%. The cross-attention fusion mechanism, through adaptive feature weight modulation, enhanced the classification accuracy to 97.53%. The fusion strategy proposed in this paper first employs HShuffleBlock for feature transformation to align dual-modal feature dimensions, then utilizes a lightweight gating network to dynamically modulate feature importance, and incorporates CBAM attention mechanism to enhance feature representation. This approach achieved optimal performance across all evaluation metrics, with a classification accuracy of 98.75%, precision of 98.82%, recall of 98.75%, and F1-score of 98.74%, representing a 1.92% improvement over the baseline feature concatenation method. Quantitative analysis indicates that the proposed fusion strategy can more effectively utilize the complementary information from dual-modal data, significantly enhancing the model’s classification performance.

### 3.2 Ablation experiment analysis

To systematically evaluate the effectiveness of key modules in the proposed model, this study conducted ablation experiments to assess the impact of the HShuffleBlock feature transformation module, CBAM attention mechanism, and lightweight gated fusion module on model performance. **Table 4** presents the experimental results for different module combinations.

**Table 4:** Ablation experiment.

HShuffleBlock	CBAM	GatedFusion	ACC (%)	Pre (%)	Rec (%)	F1 (%)	Params (M)	FLOPs (M)
			96.83 ± 0.43	97.07 ± 0.36	96.83 ± 0.43	96.78 ± 0.46	11.02	65.36
**√**			97.16 ± 0.42	97.29 ± 0.37	97.16 ± 0.42	97.15 ± 0.43	2.44	63.45
	**√**		97.33 ± 0.15	97.40 ± 0.14	97.33 ± 0.15	97.33 ± 0.15	11.12	70.12
		**√**	97.50 ± 0.20	97.60 ± 0.17	97.50 ± 0.20	97.49 ± 0.20	11.08	72.89
	**√**	**√**	98.57 ± 0.26	98.65 ± 0.24	98.57 ± 0.26	98.56 ± 0.27	11.34	76.68
**√**		**√**	97.91 ± 0.38	98.03 ± 0.32	97.91 ± 0.38	97.90 ± 0.39	2.46	72.93
**√**	**√**		98.04 ± 0.43	98.15 ± 0.38	98.04 ± 0.43	98.02 ± 0.43	2.49	69.82
**√**	**√**	**√**	**98.75 ± 0.09**	**98.82 ± 0.11**	**98.75 ± 0.09**	**98.74 ± 0.10**	**2.53**	**75.40**

√ indicates the module is included in the network architecture, while blank cells indicate the module is not used.

The bold values indicate the best performance achieved across all experimental configurations.

Initially, the baseline model (without any enhancement modules) achieved a classification accuracy of 96.83%, validating the fundamental effectiveness of multimodal fusion. Building upon this, the independent introduction of the HShuffleBlock feature transformation module improved the model accuracy to 97.16% while significantly reducing model parameters (from 11.02M to 2.44M), confirming its advantages in feature alignment and model lightweighting.

The standalone implementation of the CBAM attention mechanism increased accuracy to 97.33%, demonstrating its effectiveness in enhancing feature representation. Meanwhile, the independent adoption of the gated fusion module improved accuracy to 97.50%, indicating the positive impact of adaptive feature fusion strategies.

Further analysis revealed that the combination of HShuffleBlock and CBAM achieved an accuracy of 98.04%, while the HShuffleBlock and gated fusion combination reached 97.91% accuracy. The CBAM and gated fusion combination attained 98.57% accuracy. These results indicate synergistic effects between different modules, which complementarily enhance model performance.

Ultimately, the complete model incorporating all three modules achieved optimal performance across all evaluation metrics, with a classification accuracy of 98.75 ± 0.09% and precision of 98.82 ± 0.11%, while maintaining low computational complexity (2.53M parameters, 75.40M FLOPs). Compared to the baseline model, it demonstrated a 1.92% improvement in accuracy while reducing parameters by 77.04%, validating the advantages of the proposed method in both performance and efficiency.

Notably, the HShuffleBlock module significantly reduced model parameters and computational cost while maintaining or improving performance, which has important implications for practical applications. The experimental results demonstrate that the synergistic effect of the three key modules not only enhanced model performance but also maintained high computational efficiency, providing an effective and feasible solution for crop classification tasks.

### 3.3 Comparative analysis of attention mechanisms

To systematically evaluate the applicability of different attention mechanisms within the proposed model framework, this study compared mainstream attention mechanisms—SE (Squeeze-and-Excitation), ECA (Efficient Channel Attention), CBAM (Convolutional Block Attention Module), and CA (Coordinate Attention)—while maintaining consistent HShuffleBlock and GatedFusion modules. **Table 5** presents performance comparison results when different attention mechanisms are combined with other key modules.

**Table 5:** Performance Comparison of Different Attention Mechanisms.

Attention mechanism	ACC (%)	Pre (%)	Rec (%)	F1 (%)	Params (M)	FLOPs (M)
SE	97.65 ± 0.28	97.73 ± 0.25	97.65 ± 0.28	97.64 ± 0.28	2.47	73.84
ECA	97.42 ± 0.32	97.51 ± 0.29	97.42 ± 0.32	97.40 ± 0.33	2.45	73.26
CBAM	98.75 ± 0.09	98.82 ± 0.11	98.75 ± 0.09	98.74 ± 0.10	2.53	75.4
CA	98.23 ± 0.22	98.32 ± 0.19	98.23 ± 0.22	98.22 ± 0.23	2.58	77.16

The experimental results demonstrate that different attention mechanisms exhibit notable performance variations. SE, as a classical channel attention method, achieved an accuracy of 97.65 ± 0.28%, which is 1.1 percentage points lower than CBAM. Although ECA features a more lightweight design, it showed the weakest performance in this task with an accuracy of 97.42 ± 0.32%. CA achieved an accuracy of 98.23 ± 0.22%, ranking second after CBAM. These performance differences can be attributed to the structural characteristics of each attention mechanism: SE and ECA focus solely on the importance of features in the channel dimension and cannot capture discriminative information in the spatial domain, which presents significant limitations when processing grain images with complex spatial structures. CA achieves good position sensitivity through coordinate decoupling, but its complex structure results in higher computational overhead (77.16M FLOPs).

CBAM demonstrated a clear advantage within this research framework, which is closely related to its design features. CBAM’s cascaded channel-spatial attention design forms a complementary advantage with the HShuffleBlock feature transformation module. After feature dimension alignment, discriminative features are significantly enhanced through CBAM’s dual attention processing. In terms of feature processing mechanisms, CBAM captures global information in the channel dimension through parallel average pooling and maximum pooling paths, while CBAM’s 7×7 convolution spatial attention mechanism provides sufficient receptive field to effectively capture key regional features of grain morphology.

### 3.4 Parameter sensitivity analysis

In order to systematically assess the stability and generalization ability of the model, this study conducts an in-depth sensitivity analysis on three key training parameters: batch size, learning rate and feature dimension.

#### 3.4.1 Impact of batch size

Batch size is one of the crucial hyperparameters affecting model training effectiveness. Through comparative experiments within the range of [16, 128] (**Table 6**), optimal model performance was achieved with a batch size of 32. This phenomenon can be explained from an optimization theory perspective: excessively small batch sizes lead to high variance in gradient estimation, resulting in unstable training processes. Conversely, while larger batch sizes provide more accurate gradient estimates, they reduce the model’s sensitivity to training data, affecting its generalization performance. Furthermore, considering the characteristics of crop spectral-image data, a batch size of 32 maintains sufficient randomness while ensuring reasonable training efficiency, making it the optimal choice for practical applications.

**Table 6:** Effect of batch size on model performance.

Batch Size	Accuracy (%)	Parameters (M)	FLOPs (M)
16	97.20 ± 0.15	2.53	75.4
32	98.75 ± 0.09	2.53	75.4
64	98.10 ± 0.12	2.53	75.4
128	97.50 ± 0.18	2.53	75.4

#### 3.4.2 Impact of learning rates

As a core parameter of the optimizer, learning rate directly influences model convergence performance. This study employed a hierarchical learning rate strategy, conducting detailed tests within the range of [0.0001, 0.01]. As shown in **Table 7**, a base learning rate of 0.001 achieved optimal performance. Specifically, the pre-trained feature extraction branch utilized 0.1 times the base learning rate, while the newly added fusion module used the base learning rate. This strategy maintained the stability of pre-trained weights while allowing the fusion module to rapidly adapt to specific tasks. When the learning rate was reduced to 0.0001, model convergence was slow and performance was limited. When increased to 0.01, model performance declined significantly, indicating that excessive learning rates disrupt the effective features captured by the pre-trained model, hindering stable training.

**Table 7:** Effect of learning rate on model performance.

Learning Rate	Accuracy (%)	Parameters (M)	FLOPs (M)
0.0001	96.90 ± 0.21	2.53	75.4
0.001	98.75 ± 0.09	2.53	75.4
0.05	97.80 ± 0.14	2.53	75.4
0.01	95.50 ± 0.25	2.53	75.4

#### 3.4.3 Effects of characterization dimensions

Feature dimension is a crucial factor determining model expressiveness and computational efficiency. This study analyzed the impact of common feature space dimensions within the range of [128, 512]. As shown in **Table 8**, a dimension of 384 achieved optimal balance between performance and efficiency. While smaller feature dimensions offered lower computational complexity, they limited the model’s expressive capacity, leading to performance degradation. Conversely, excessive dimensions not only increased computational overhead but potentially introduced redundant information, risking overfitting. Notably, experiments revealed a significant correlation between feature dimension and gated fusion module performance, with the 384-dimension configuration providing adequate yet non-redundant information representation space for attention mechanisms and feature fusion.

**Table 8:** Impact of feature dimensions on model performance.

Feature Dim	Accuracy (%)	Parameters (M)	FLOPs (M)
128	96.80 ± 0.19	2.1	65.2
256	97.90 ± 0.15	2.3	70.1
384	98.75 ± 0.09	2.53	75.4
512	98.20 ± 0.13	2.8	82.3

Through detailed parameter sensitivity analysis, we determined the optimal model configuration for practical applications: batch size of 32, base learning rate of 0.0001, and feature dimension of 384. This configuration not only demonstrates performance advantages but also maintains favorable computational efficiency. The experimental results further indicate that the proposed model maintains stable performance across a wide range of parameters, exhibiting robust characteristics that hold significant practical implications for crop classification task deployment.

### 3.5 Analysis of improvement effects

**Figure 14:**
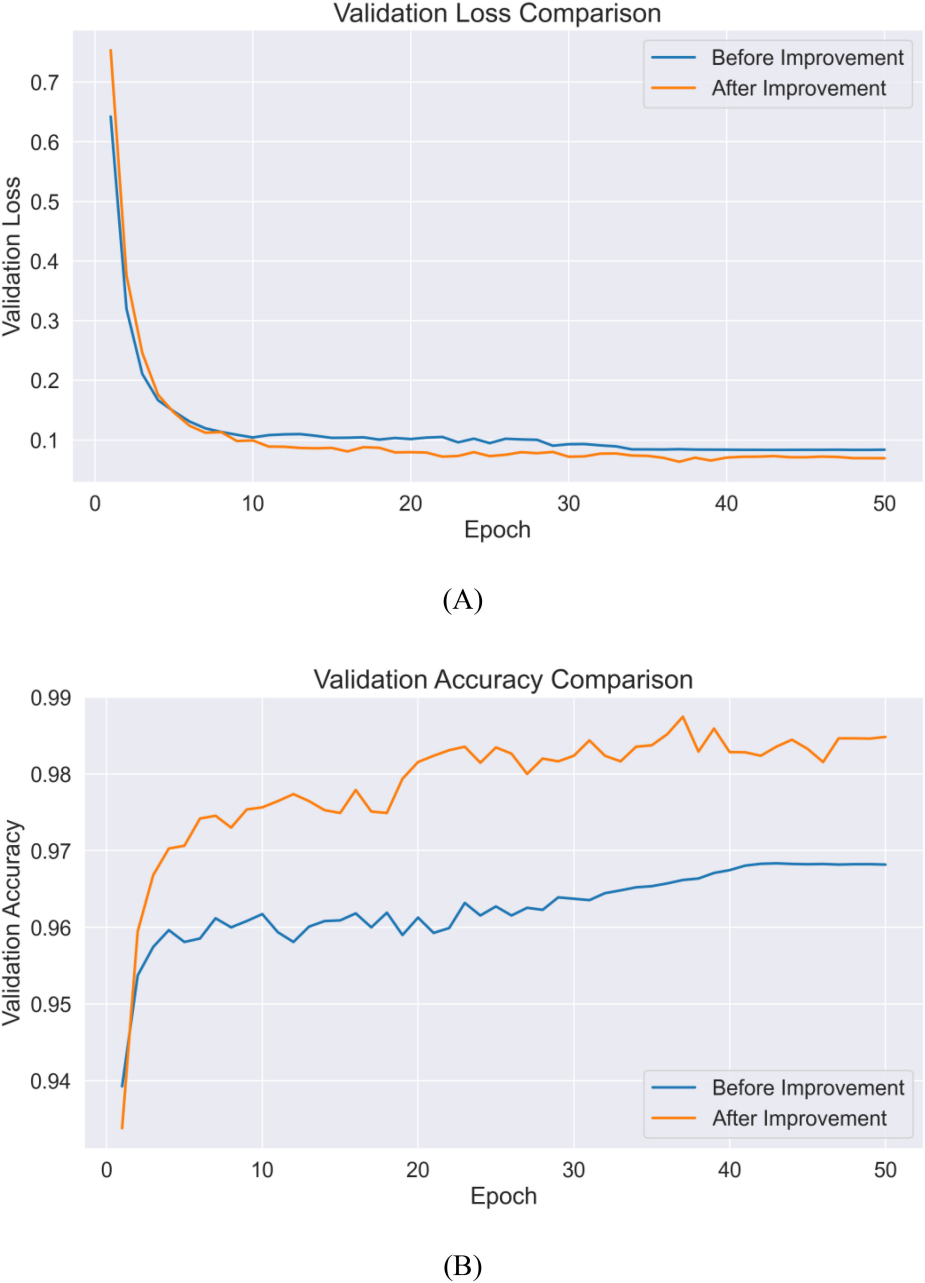
Comparison of loss values and validation accuracy before and after improvement **(A)** validation loss **(B)** validation accuracy.

**Figure 15:**
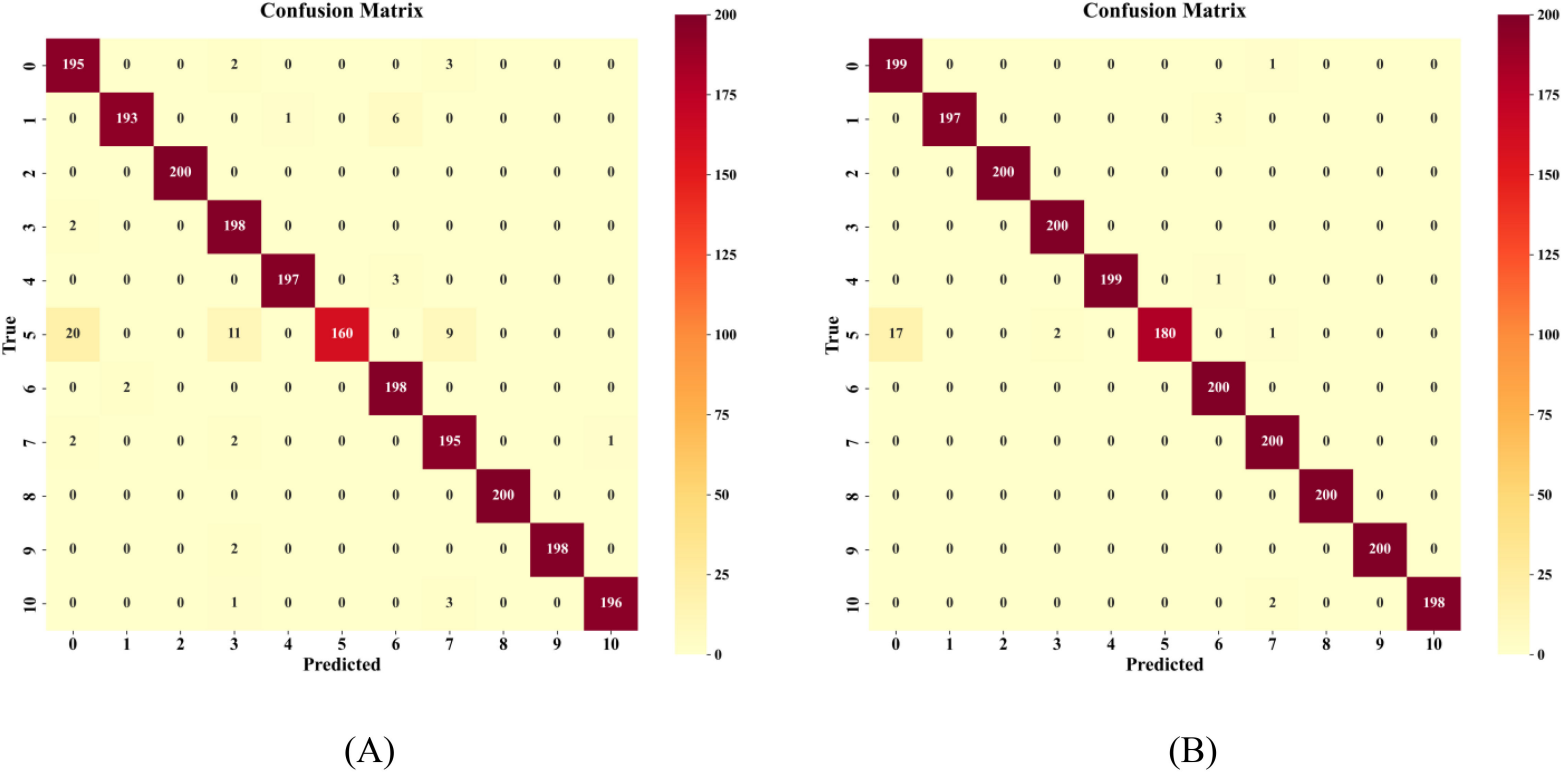
Comparison of Confusion Matrices **(A)** Before improvement **(B)** After improvement.

To comprehensively evaluate the effectiveness of the proposed improvement strategies, this section presents a comparative analysis focusing on model convergence performance and classification results. **Figure 14** illustrates the performance trends on the validation set before and after improvements, while **Figure 15** provides detailed confusion matrix comparison results.

In terms of model convergence characteristics, the improved model achieved significant enhancement in both convergence speed and final performance. As shown in **Figure 14A**, during the initial training phase (1–10 epochs), both versions demonstrated rapid learning capabilities, with validation accuracy quickly rising above 95%. However, the improved model exhibited stronger feature extraction capabilities, achieving a validation accuracy of 98.2% by epoch 20, an increase of nearly 2 percentage points compared to the pre-improvement version. This enhancement primarily stems from the introduction of the HShuffleBlock, which strengthens feature representation through grouped linear transformations and channel reorganization. The comparison of loss values (**Figure 14B**) reveals that the improved model’s validation loss converged to 0.063, while the pre-improvement model fluctuated between 0.080 and 0.085, validating the effectiveness of the CBAM attention mechanism in suppressing noise features and highlighting discriminative features.

Regarding classification performance, the improvement strategies significantly enhanced the model’s variety recognition capabilities. In the pre-improvement model (**Figure 15A**), notable confusion occurred between JD50 and JD209, as well as between JD626 and JD83, due to high similarities in their spectral and morphological features. In the improved model (**Figure 15B**), the introduction of the gated fusion module achieved adaptive weight allocation for different modal features, significantly enhancing the model’s ability to distinguish similar varieties. For instance, although JD50 still had 20 misclassified samples, the confusion pattern became more concentrated, indicating better capture of subtle discriminative features. Notably, six varieties including JD505 and JD27 achieved 100% recognition accuracy, confirming the effectiveness of the proposed multimodal feature fusion strategy in integrating spectral and spatial information.

Furthermore, the improved model’s high stability and generalization capability result from the synergistic effect of multiple modules: HShuffleBlock enhanced feature expression through feature reorganization, the CBAM mechanism achieved adaptive feature weighting, and the gated fusion module ensured optimal integration of multimodal information. This multi-level feature enhancement and fusion strategy effectively addressed the common feature similarity issues in crop variety recognition.

The experimental results demonstrate that the proposed improvement strategies not only achieved significant enhancement in quantitative metrics but, more importantly, mechanistically strengthened the model’s perception and representation capabilities for crop features, providing a reliable technical solution for precise crop variety recognition.

### 3.6 Correlation between morphophysiological characteristics and model performance

In single-modality experiments, RGB image classification demonstrated higher recognition accuracy for varieties with significant morphological differences. Distinct morphological variations exist among different maize varieties. The orange appearance of JiDan505 and LY9915 forms a stark contrast with other yellow varieties, providing significant features for image recognition. Similarly, the morphological differences between the elongated elliptical shape of JiDan50 and the round appearance of JiDan209 contribute to the model’s ability to differentiate between them. However, variety pairs with similar morphological features still present recognition challenges. JiDan27 and JiDan209 exhibit high similarities in shape and color. These similarities limit the accuracy of single-modality RGB image recognition.

Spectral data analysis indicates that differences in physicochemical composition among varieties can be manifested through spectral characteristics. As shown in **Figure 3E**, the spectral curves of various varieties display notable differences, particularly in the visible light and near-infrared regions. These differences are closely related to the physicochemical composition characteristics displayed in **Table 3**. For instance, JiDan953 (77.33%) and JiDan407 (76.60%), which have the highest crude starch content, exhibit characteristic spectral responses in the near-infrared region; the spectral differences between JiDan83 with the highest crude protein content (10.92%) and JiDan27 (8.46%) and ZD958 (8.47%) with the lowest crude protein content provide important evidence for spectral-based identification. Similarly, LY9915 with the highest crude fat content (4.99%) exhibits distinctly different reflectance characteristics in specific bands compared to JiDan407 with the lowest content (3.23%).

Combined analysis of morphological and physicochemical characteristics suggests that variety pairs with similar morphology but significant differences in physicochemical composition best demonstrate the advantages of multimodal fusion. For example, misclassification cases between JiDan626 and JiDan83 decreased from 6 to 3 instances; these two varieties have similar morphology but significant differences in protein content (8.66% *vs*. 10.92%). JiDan209 (aspect ratio 1.17) and JiDan50 (aspect ratio 1.60) show obvious differences in shape but also differ in physicochemical indicators (crude starch content of 68.50% and 72.60%, respectively). Similarly, while ZD958 and JiDan27 have similar crude protein content (8.47% and 8.46%, respectively), their morphological features differ significantly (aspect ratios of 1.28 and 1.17, respectively), which also aids the model in accurate differentiation. The fusion model successfully improved recognition accuracy by comprehensively considering these multidimensional features.

Changes in the confusion matrix reflect three advantageous scenarios of multimodal fusion: (1) For varieties with unique morphological features, such as the orange-colored JiDan505, the fusion model enhances the weight of morphological information; (2) For varieties with specific physicochemical compositions, such as the high-protein JiDan83, the model more effectively utilizes spectral features; (3) For variety pairs that are morphologically similar but physicochemically different, or physicochemically similar but morphologically different, the fusion strategy significantly improves recognition accuracy by balancing the contribution of both types of features.

This multimodal fusion strategy based on morphophysiological characteristics is key to the 98.75% high recognition accuracy achieved by the model proposed in this study. Through an in-depth understanding of the differences in morphological and physicochemical characteristics among maize varieties, DualCMNet not only provides an efficient and reliable method for precise identification of maize varieties but also offers a referenceable technical framework for variety recognition tasks of other crops.

## 4 Discussion

The proposed dual-branch deep learning framework has achieved significant results in maize variety identification. The experimental results reveal the inherent mechanisms of multimodal feature fusion and its impact on recognition performance. In single-modality experiments, the RGB image-based MobileNetV3 and hyperspectral data-based one-dimensional convolutional network achieved accuracy rates of 92.00% and 92.42%, respectively, reflecting the complementary nature of these two modalities in variety identification. RGB images primarily capture the morphological characteristics of kernels (such as size, shape, and texture), while hyperspectral data records the physicochemical properties, particularly the molecular vibration and chemical composition information contained within 2,151 spectral bands. This complementarity aligns with the multimodal information theory proposed by Liu et al. (2023), which suggests that information carried by different modalities exhibits partially overlapping and partially complementary characteristics. Through appropriate fusion strategies, information gain can be maximized, ultimately improving classification accuracy to 98.75%.

During the feature fusion process, each module’s design played a unique role. The HShuffleBlock significantly enhanced feature fusion through grouped linear transformation and channel shuffling, improving accuracy from 96.83% to 97.16%. This improvement primarily stems from the channel shuffling mechanism, which strengthened information interaction between different features. The design was inspired by ShuffleNet proposed by Ma et al. (2018) and research on grouped convolutions by Zhang et al. (2018); however, this study optimized their application for multimodal fusion scenarios. Experimental results demonstrate that this structure performs effectively in crop variety identification tasks, maintaining feature expression capability while significantly reducing the number of parameters. Experimental results regarding feature dimensionality indicate that a 384-dimensional feature space achieves optimal balance by providing sufficient capacity for multi-scale feature expression while avoiding redundancy from excessive dimensionality. The introduction of the CBAM module further improved accuracy, where channel attention learned dependencies between feature channels, adaptively emphasizing more discriminative band features, enabling precise differentiation between varieties with similar spectra (such as JD50 and JD209). Spatial attention enhanced local morphological feature expression, achieving fine-grained feature capture. According to Woo et al. (2018), attention mechanisms operating across both channel and spatial dimensions are complementary, jointly enhancing feature discriminability. In the specific context of crop identification, this dual attention mechanism proves particularly effective, as different grain varieties typically exhibit subtle differences in specific spatial regions and specific spectral bands. Building upon this, the lightweight gating fusion mechanism designed in this study reduced feature dimensionality to 1/8 of the original and employed a simplified two-layer perceptron structure. This mechanism learns a single gate value to dynamically adjust the proportion of multiplicative interaction and additive combination, ensuring adaptive feature fusion while significantly reducing computational overhead. Ablation experiments verified the effectiveness of this design, as removing the gating mechanism decreased model performance by 1.25 percentage points, indicating the crucial role of lightweight gated fusion in balancing computational efficiency and model expressiveness. Research by Zadeh et al. (2018) indicates that multiplicative interaction can capture non-linear correlations between modalities, while additive combination better preserves the independent information of each modality. The gating mechanism in this study dynamically balances these two operations, enabling the model to adaptively select the optimal fusion method according to the characteristics of the input data.

The staged training strategy proved particularly effective, with accuracy improving by 2.1% after the 14th epoch. This strategy preserved general visual feature extraction capabilities by freezing pre-trained layers in the initial stage, then allowed model optimization through differentiated learning rates (0.01× for pre-trained layers, 0.1× for fusion layers) while maintaining basic features and optimizing task-specific feature expression. Smaller batch sizes provided more frequent parameter updates, facilitating the model’s exploration of optimal feature combinations, explaining why larger batches led to performance degradation. These findings suggest that model performance improvements result from the synergistic effects of feature extraction, attention mechanisms, lightweight gated fusion, and training strategies.

From an application perspective, this study contributes by providing a solution that balances accuracy and efficiency. With the development of smart agriculture, seed identification systems need to operate on resource-constrained devices such as portable analyzers or edge computing equipment. Agricultural field applications impose strict limitations on model size and energy consumption to accommodate the computational capabilities and power constraints of embedded devices (Chenglong et al., 2022). The framework proposed in this study has only 2.53M parameters, largely meeting lightweight requirements and making the model more suitable for deployment on resource-constrained embedded devices while maintaining high recognition accuracy. Furthermore, sensitivity analysis of the model to different batch sizes and learning rates indicates that the framework exhibits good stability when parameters change, which is crucial for practical deployment.

The proposed method has certain limitations. Regarding environmental adaptability, since the experimental data collection was conducted under standardized conditions, the model’s performance under complex lighting and background conditions remains to be verified. To address this limitation, future research could incorporate data augmentation techniques to simulate samples under varying lighting and background conditions, or implement adversarial training strategies to enhance model adaptability to environmental changes. Furthermore, constructing large-scale datasets comprising samples collected under diverse environmental conditions represents an effective approach to improving model generalization. Regarding computational resources, despite achieving a relatively low parameter count (2.53M) compared to existing methods, the 75.40M FLOPs still presents optimization opportunities and may restrict model deployment on edge devices. Solutions can be developed in two directions: on one hand, band selection algorithms could be applied to retain only the most contributory spectral bands for variety identification, thereby reducing input dimensionality; on the other hand, more efficient network architectures could be explored, such as introducing lightweight convolution operations or employing model compression techniques to further decrease computational complexity. Although the current feature fusion mechanism significantly enhances model performance, there remains scope for improvement in distinguishing highly similar varieties (e.g., JiDan50 and JiDan209). This is primarily because the differences in spectral and morphological features between these varieties are extremely subtle, and existing attention mechanisms may inadequately capture these minute variations. Potential improvements could include developing adaptive weight allocation mechanisms for key spectral bands or introducing multi-scale feature analysis methods to enhance perception of subtle differences. Notably, the dual-branch architecture proposed in this study demonstrates good scalability, and this framework based on spectral-spatial feature fusion could be extended to other crop variety identification tasks, such as rice and wheat. This is because different crop varieties similarly possess unique spectral and morphological characteristics, and the feature reorganization, attention enhancement, and lightweight gated fusion strategies designed in this research can effectively capture and integrate these features. Addressing these issues, future research could focus on: 1) Feature selection based on spectral information content to reduce feature redundancy; 2) Refinement of feature weight allocation mechanisms to enhance model sensitivity to subtle varietal differences; 3) Optimization of gate structure design to further improve computational efficiency. These optimization directions hold significant importance for enhancing the practicality of crop variety identification technology.

## 5 Conclusion

This study presents a novel dual-branch deep learning framework, DualCMNet, which achieves 98.75% accuracy in identifying 11 maize varieties by integrating hyperspectral data with RGB image information. The research demonstrates that multimodal data fusion effectively extracts both physicochemical properties and morphological features of maize kernels, providing an accurate and reliable solution for crop variety identification.

The framework incorporates three key modules: feature recombination, attention enhancement, and lightweight gated fusion, enabling effective integration of spectral-spatial features. The HShuffleBlock achieves efficient feature recombination through grouped linear transformation and channel shuffling, promoting thorough feature fusion and enhancing the model’s feature extraction capabilities. The CBAM attention mechanism significantly improves the model’s perception of key features, enabling precise discrimination between varieties with similar spectral and morphological characteristics. The lightweight gated fusion module dynamically adjusts feature weights through learning a single gate value, achieving optimal balance between computational efficiency and fusion performance.

Furthermore, the adopted staged training strategy effectively balances the retention of pre-trained knowledge and the learning of new task features through differentiated learning rates. Compared to unimodal approaches, the proposed multimodal fusion framework demonstrates significant improvements across key metrics, validating its technical advantages in crop variety identification tasks.

In conclusion, this research not only provides an efficient and accurate solution for maize variety identification but also presents new perspectives for the application of computer vision technology in agriculture.

## Data Availability

The data analyzed in this study is subject to the following licenses/restrictions: The raw data supporting the conclusions of this article will be made available by the authors, without undue reservation. Requests to access these datasets should be directed to CB, chunguangb@jlau.edu.cn.
